# *OsWRKY67* positively regulates blast and bacteria blight resistance by direct activation of *PR* genes in rice

**DOI:** 10.1186/s12870-018-1479-y

**Published:** 2018-10-26

**Authors:** Qing Liu, Xia Li, Shijuan Yan, Ting Yu, Jianyuan Yang, Jingfang Dong, Shaohong Zhang, Junliang Zhao, Tifeng Yang, Xingxue Mao, Xiaoyuan Zhu, Bin Liu

**Affiliations:** 1 Guangdong Key Laboratory of New Technology in Rice Breeding, Rice Research Institute, Guangdong Academy of Agricultural Sciences, Guangzhou, 510640 China; 2 School of Plant Science and Technology, Huazhong Agricultural University, Wuhan, 430070 China; 3 Agro-biological Gene Research Center, Guangdong Academy of Agricultural Sciences, Guangzhou, 510640 China; 4 Guangdong Key Laboratory of New Technology in Plant Protection, Plant Protection Research Institute, Guangdong Academy of Agricultural Sciences, Guangzhou, 510640 China

**Keywords:** *OsWRKY67*, Leaf blast, Panicle blast, Bacterial blight, Salicylic acid

## Abstract

**Background:**

WRKY proteins are one of the largest gene families and are well-known for their regulatory roles in many aspects of plant development, including plant response to both biotic and abiotic stresses. Although the roles of WRKY proteins in leaf blast resistance have been well-documented in rice, their functions in panicle blast, the most destructive type of blast disease, are still largely unknown.

**Results:**

Here, we identified that the transcription of *OsWRKY67* was strongly activated by leaf and panicle blast infection. *OsWRKY67* is ubiquitously expressed and sub-localized in the nucleus. Rice plants overexpressing *OsWRKY67* showed quantitatively enhanced resistance to leaf blast, panicle blast and bacterial blight. In contrast, silencing of *OsWRKY67* increased the susceptibility to blast and bacterial blight diseases. RNA-seq analysis indicated that *OsWRKY67* induces the transcription of a set of defense-related genes including the ones involved in the salicylic acid (SA)-dependent pathway. Consistent with this, the *OsWRKY67-*overexpressing plants accumulated higher amounts of endogenous SA, whereas lower endogenous SA levels were observed in *OsWRKY67-*silenced plants relative to wild-type *Nipponbare* plants before and after pathogen attack. Moreover, we also observed that *OsWRKY67* directly binds to the promoters of *PR1a* and *PR10* to activate their expression.

**Conclusions:**

These results together suggest the positive role of *OsWRKY67* in regulating rice responses to leaf blast, panicle blast and bacterial blight disease. Furthermore, conferring resistance to two major diseases makes it a good target of molecular breeding for crop improvement in rice.

**Electronic supplementary material:**

The online version of this article (10.1186/s12870-018-1479-y) contains supplementary material, which is available to authorized users.

## Background

Rice blast and bacterial blight diseases are two of the leading causes of rice yield loss all over the world [1]. Nowadays, it is a common issue that the rice cultivars always exhibit a short-life span of disease resistance and how to extend the life span of disease resistance is the priority for rice improvement. Employment of host resistance is deemed as the most effective way to address this issue [2]. Host resistance of plants consists of both qualitative (complete) resistance and quantitative (partial) resistance [3, 4]. Quantitative resistance conferred by multiple genes is considered to be more broad-spectrum and durable than qualitative resistance mediated by *R* genes, based on its non-race specificity [5]. Therefore, quantitative resistance is the favored strategy for sustainable control of plant disease. The quantitative resistance of rice is the result of the comprehensive effect of various kinds of genes, including transcription factors (TF) such as WRKYs.

WRKY TFs are one of the largest gene families and are well-known for their regulatory roles in almost every aspect of plant development, including plant response to both biotic and abiotic stresses [6]. To date, many WRKY proteins have been demonstrated to associate with various defense responses in *Arabidopsis*, tobacco and barley [7–13]. In rice, more than 100 WRKY genes have been identified [14], and several of these genes (e.g. *OsWRKY13*, *OsWRKY31*, *OsWRKY45*, *OsWRKY53*, *OsWRKY71*, *OsWRKY89* and *OsWRKY76*) have been confirmed to positively regulate rice resistance to blast or bacterial blight via transgenic technology [15–22]. Moreover, *OsWRKY62* negatively regulates the basal and *Xa21*-mediated resistance to bacterial blight [23], and *OsWRKY28* is linked to a negative role in blast disease resistance [24].

Despite these results, the regulatory roles of most of the WRKY genes in defense response are still largely unknown. Rice blast disease can be differentiated as leaf blast and panicle blast based on the location of infection in a plant. Panicle blast is more destructive than leaf blast because of its more direct relationship to yield loss. Inconsistent findings of plant response in leaf blast and panicle blast resistance suggest that the mechanisms of rice response to leaf blast and panicle blast likely differ to some extent [25–27]. However, majority of studies on WRKY genes in rice blast resistance reported so far have focused on leaf blast except for *OsWRKY45*, which confers durable resistance to panicle blast [28]. Thus, we still have very limited knowledge about the roles of other WRKY genes in panicle blast resistance in rice.

To investigate the functions of WRKY genes on both leaf and panicle blast resistance, we performed a genome-wide differential gene expression analysis via microarray of a blast resistant advanced backcross line that was infected with leaf and panicle blast. Our results showed that most of the WRKY genes differentially expressed with rice blast infection, implying their crucial roles in regulating rice blast resistance. Among the differentially expressed WRKY genes, we identified that *OsWRKY67* (*Os05g0183100*) was strongly activated by leaf blast and panicle blast infection. The gene overexpression and RNAi silencing experiments suggest that *OsWRKY67* positively regulates leaf blast, panicle blast and bacterial blight resistance in rice. Expression profiling in *OsWRKY67*-overexpressing plants indicates that subsets of defense-related genes were divergently regulated by *OsWRKY67.* Further analysis also suggests that *OsWRKY67* confers disease resistance through the activation of the salicylic acid (SA) signaling pathway and directly targets pathogenesis-related (PR) proteins 1a (*PR1a*) and 10 (*PR10*).

## Results

### Transcription of *OsWRKY67* is activated by blast inoculation

**Fig. 1:**
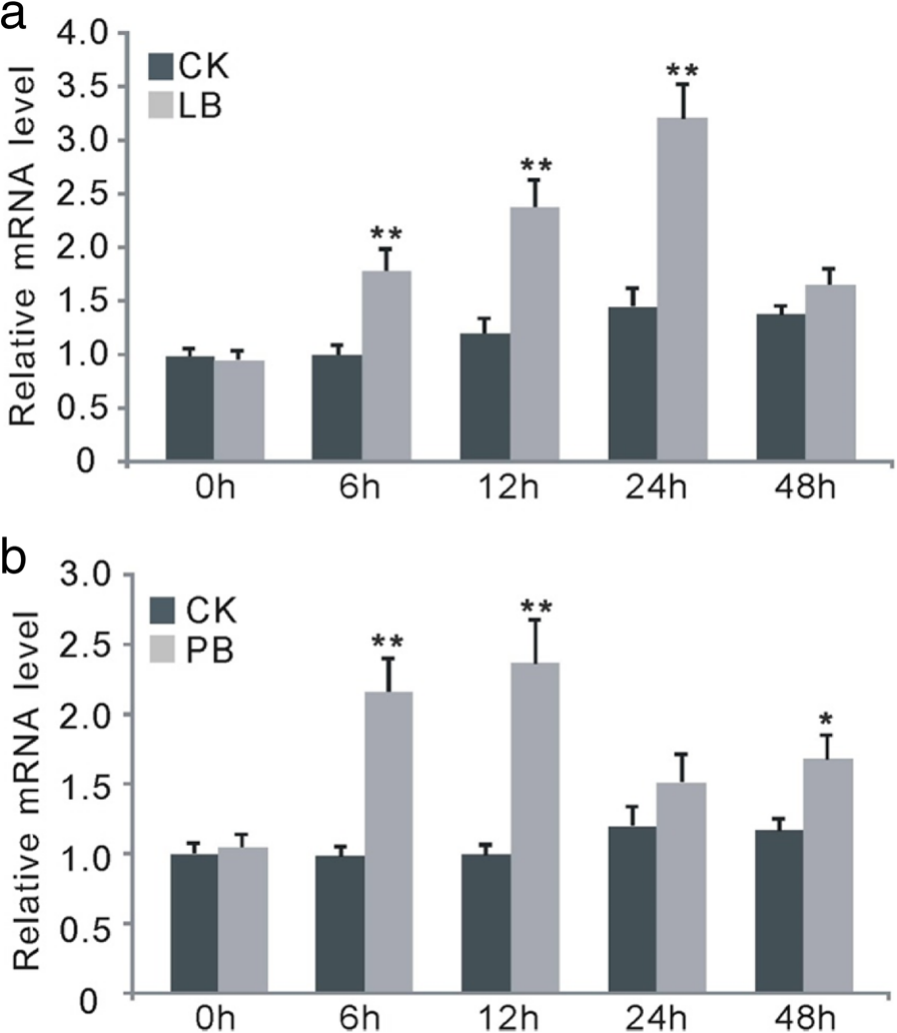
The expression pattern of *OsWRKY67* after blast fungus infection. The values are means ± standard deviations (SDs) of three biological replicates, and asterisks represent significant differences relative to the water treatment (0 h) (*t*-test, **P < 0.01 and *P < 0.05). **a** The transcription of *OsWRKY67* was activated by leaf blast infection. LB = leaf blast. **b** The transcription of *OsWRKY67* was activated by panicle blast infection. PB = panicle blast

Our previous microarray analysis for excavating genes that were regulated by blast infection showed that the expression of *OsWRKY67* was remarkably activated by both leaf blast and panicle blast inoculation (Additional file 1: Table S1). Here, to confirm this result, the expression patterns of *OsWRKY67* were also analyzed in *Nipponbare* plants after infection with leaf blast and panicle blast, and real-time PCR was conducted at 6, 12, 24 and 48 h after inoculation. Our results indicated that the transcription levels of *OsWRKY67* were remarkably activated at 6, 12 and 24 h in both leaves and panicles after pathogen inoculation, then slightly declined in leaves but remained highly expressed in panicles at 48 h (Fig. 1). These results together suggest the important role of *OsWRKY67* in regulating blast disease resistance in rice.

### *OsWRKY67* is ubiquitously expressed and is sub-localized in the nucleus

**Fig. 2:**
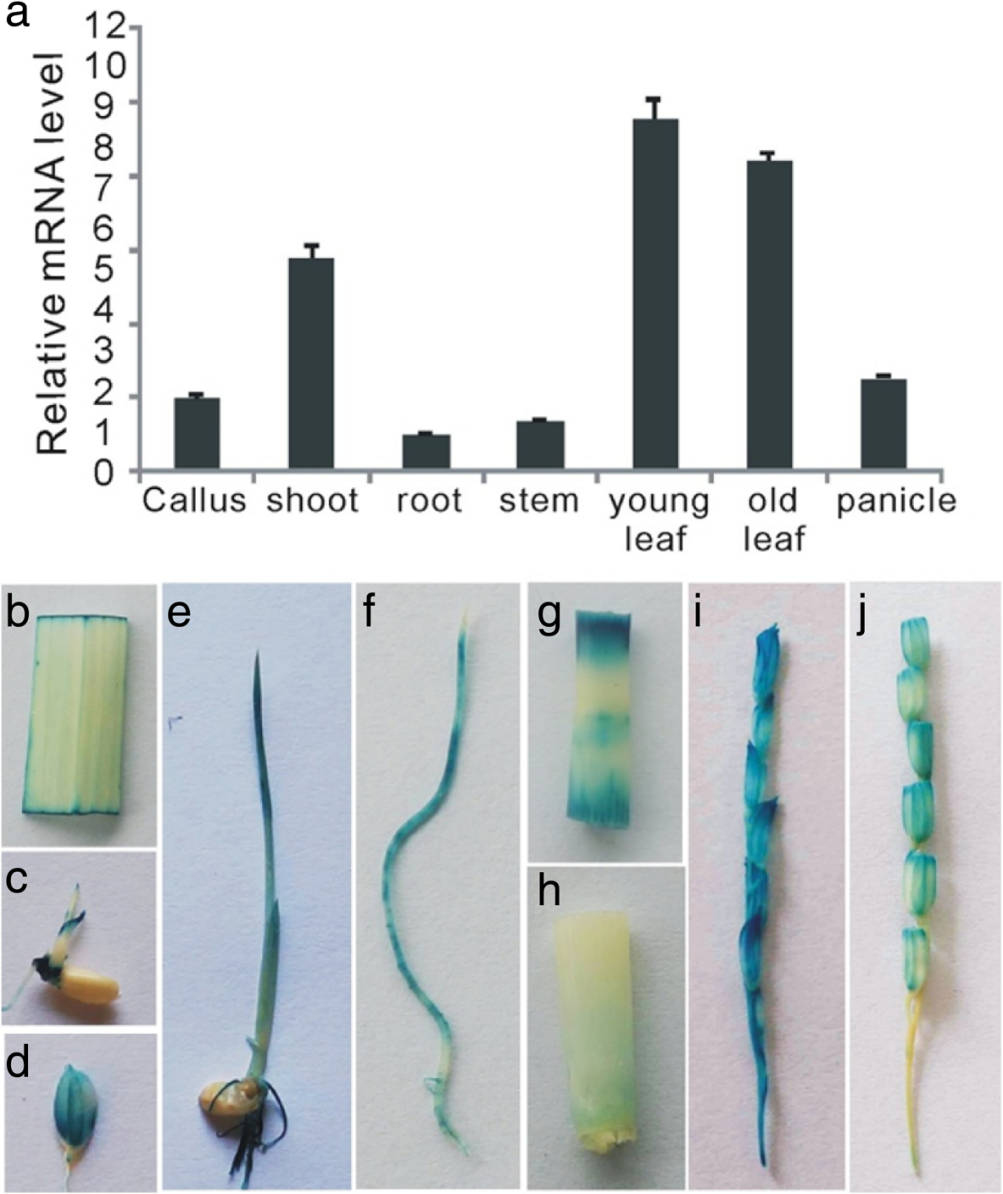
The spatiotemporal expression of *OsWRKY67* in *Nipponbare* plants. **a** Relative expression levels of *OsWRKY67* in different *Nipponbare* tissues. Young leaf, leaf at the three- to four-leaf stage; old leaf, leaf at the booting stage. The values are means ±SDs of three biological replicates. **b-j** *GUS* staining analysis of *OsWRKY67* in different *Nipponbare* tissues. **b** leaf at the booting stage; **c** sprouting stage; **d** hull; **e** one-week old seedling; **f** root at the booting stage; **g** the second node at the booting stage; **h** the first node at the booting stage; **i** panicle at the booting stage; **j** panicle at the heading stage

To determine the spatiotemporal expression of *OsWRKY67* in rice, real-time PCR analysis was conducted in different *Nipponbare* tissues and the result revealed that *OsWRKY67* was expressed in all rice tissues inspected, with the highest level in leaf tissues (Fig. 2a). We also fused the promoter of *OsWRKY67* to the β-glucuronidase (GUS) gene to generate *OsWRKY67*_*pro*_::GUS transgenic plants in *Nipponbare*, and the GUS staining analysis was also performed in different tissues of these transgenic plants. The GUS signal was detected in the leaf, bud, node, spikelet hull, one-week old seedling, root, panicles at the booting stage and panicles at the heading stage (Fig. 2b-j). These observations supported the results from the qRT-PCR, suggesting that *OsWRKY67* is expressed in different tissues during the entire rice life cycle.

To investigate the subcellular localization of *OsWRKY67*, the CDS sequence of *OsWRKY67* was fused with the green fluorescence protein (GFP) which is controlled by the CaMV 35S promoter. We transiently expressed the GFP-OsWRKY67 fusion protein and the control protein (empty GFP protein) in onion epidermal cells. The results showed that the GFP signal was localized in the nucleus of the cells transfected with the fusion protein, whereas the GFP signal in the control protein was universally distributed in the nucleus and cytoplasm (Additional file 2: Figure S1a). To further validate this result, the fused GFP-OsWRKY67 protein was also expressed transiently in rice protoplasts and these results also illustrated the nuclear localization of OsWRKY67 protein (Additional file 2: Figure S1b).

### Overexpression of *OsWRKY67* enhances resistance to blast and bacterial blight in rice

**Fig. 3:**
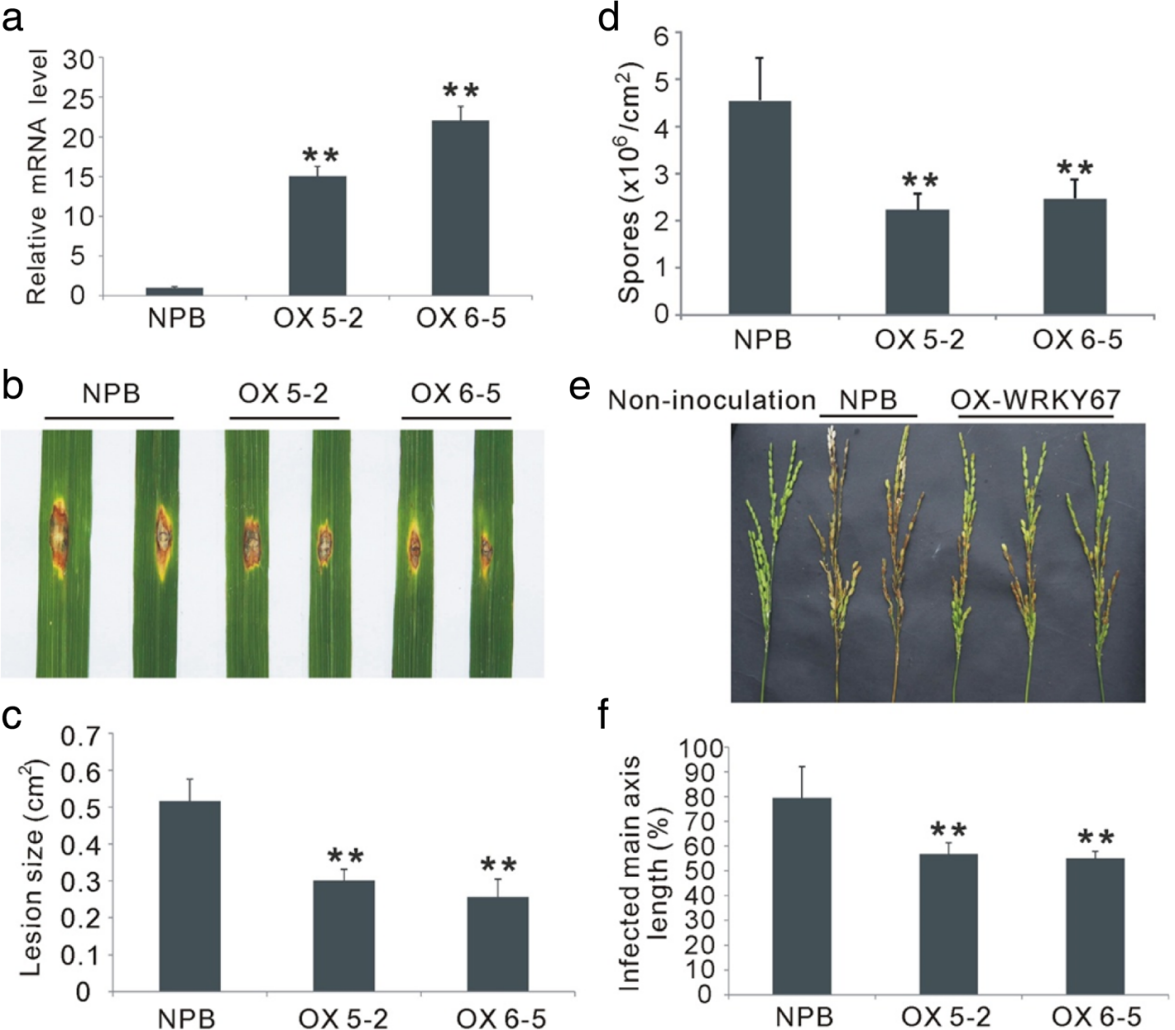
Phenotypes of the *OsWRKY67* overexpressing (OX-WRKY67) plants infected with blast fungus. The asterisks represent significant differences relative to *Nipponbare* plants (*t*-test, **P < 0.01). **a** Relative transcription levels of *OsWRKY67* in wild-type *Nipponbare* (NPB) and OX-WRKY67 plants. The values are mean ± SD of three biological replicates. **b** The OX-WRKY67 plants exhibited enhanced leaf blast resistance after inoculation with GD08-T13 using the punch method. **c** Relative lesion size in *Nipponbare* and OX-WRKY67 plants after infection by leaf blast. The values are mean ± SD of twelve biological replicates. **d** Numbers of spores produced on the *Nipponbare* and OX-WRKY67 plants after punch inoculation. The values are means ± SDs of six biological replicates. **e** The OX-WRKY67 plants exhibited enhanced panicle blast resistance after inoculation with GD08-T13. **f** The infected main axis length in *Nipponbare* and OX-WRKY67 plants after infection by panicle blast. The values are means ± SDs of twenty biological replicates

To confirm the function of *OsWRKY67* in disease resistance in rice, we produced the *OsWRKY67* overexpressing (OX-WRKY67) plants in *Nipponbare* which are susceptible to blast and *Xoo* diseases. Twelve independent transgenic lines were generated and the OX-WRKY67 plants showed a dwarfed phenotype relative to wild-type *Nipponbare* plants. The tiller numbers of OX-WRKY67 plants were also significantly lower than that of wild-type plants (Additional file 3: Figure S2). Two independent homozygous lines (OX 5–2 and OX 6–5) with high transcription levels of *OsWRKY67* were selected for disease evaluation (Fig. 3a; Additional file 4: Figure S3). In the spray inoculation experiment, the OX-WRKY67 plants exhibited fewer susceptible-type lesions compared with wild-type *Nipponbare* plants (Additional file 5: Figure S4). To further validate the role of *OsWRKY67* in leaf resistance, we evaluated leaf blast resistance of the OX-WRKY67 plants by inoculation with the same blast isolate using punch method. As expected, the lesions on the leaves of OX-WRKY67 plants were smaller than that on the leaves of *Nipponbare* plants (Fig. 3b, c). Moreover, the spores in the infected OX-WRKY67 leaves were also fewer than that in the wild-type *Nipponbare* leaves (Fig. 3d). The two over-expressed lines, OX 5–2 and OX 6–2, also showed enhanced resistance to GD08-T13 at the heading stage, with the infected main axis length ranging from 55 to 56.8%, whereas the infected main axis length was 79.5% for *Nipponbare* plants (Fig. 3e, f).

The OX-WRKY67 plants also showed significantly enhanced resistance to *Xoo* race 4 (*P* < 0.01), with lesion lengths ranging from 4.73 to 4.21 cm versus 13.78 cm for the wild type *Nipponbare* plants (Additional file 6: Figure S5a, b). The number of bacteria on OX-WRKY67 plants was much lower than that on susceptible control *Nipponbare* at 12 and 16 days after infection (Additional file 6: Figure S5c). These results together suggest that overexpression of *OsWRKY67* enhances resistance to blast and bacterial blight in rice.

### Silencing of *OsWRKY67* increases susceptibility to blast and bacterial blight in rice

**Fig. 4:**
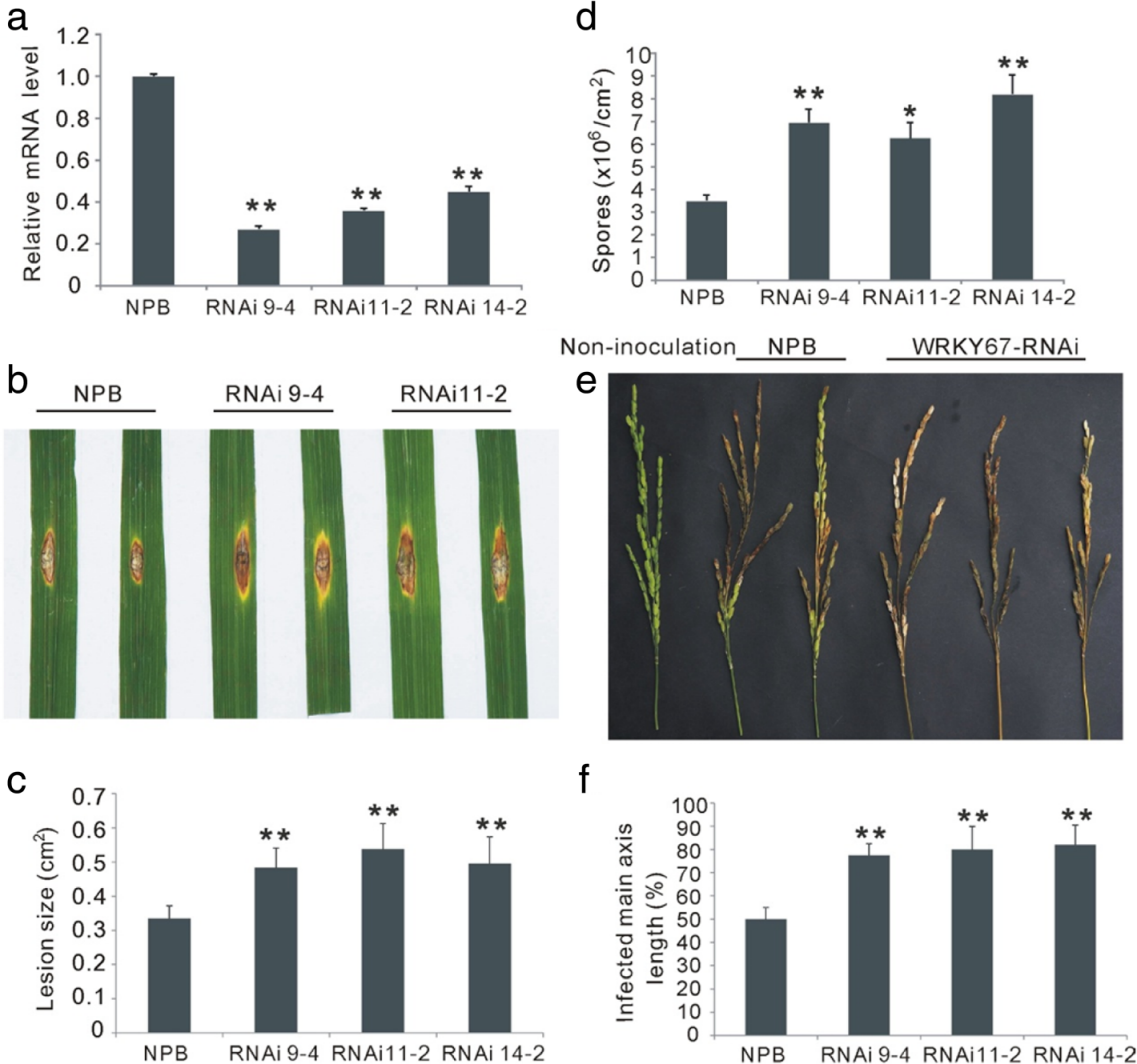
Phenotypes of the *OsWRKY67*-silenced (WRKY67-RNAi) plants infected with blast fungus. The asterisks represent significant differences relative to wild-type *Nipponbare* (NPB) plants (*t*-test, **P < 0.01 and *P < 0.05). **a** Relative transcription levels of *OsWRKY67* in *Nipponbare* and WRKY67-RNAi plants. The values are means ± SDs of three biological replicates. **b** Silencing of *OsWRKY67* increased susceptibility to leaf blast infection using punch method. **c** Relative lesion size in *Nipponbare* and WRKY67-RNAi plants after leaf blast infection. The values are means ± SDs of twelve biological replicates. **d** Numbers of spores produced on *Nipponbare* and WRKY67-RNAi plants after punch inoculation. The values are means ± SDs of six biological replicates. **e** Silencing of *OsWRKY67* increased susceptibility to panicle blast in rice. **f** The infected main axis length in *Nipponbare* and WRKY67-RNAi plants after panicle blast inoculation. The values are means ± SDs of twelve biological replicates

**Fig. 5:**
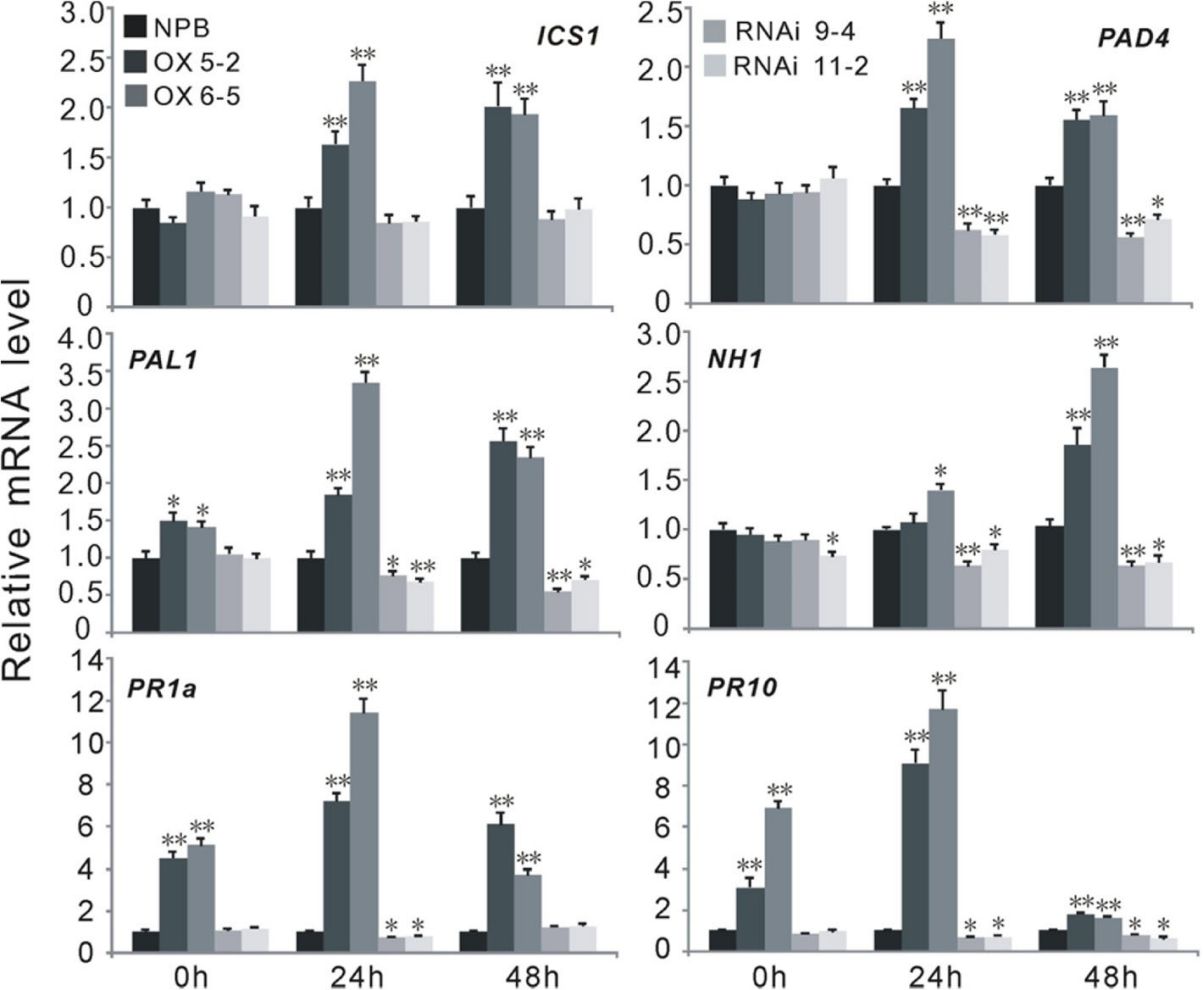
*OsWRKY67* regulates the transcription of genes involved in the salicylic acid (SA) signaling pathway. The wild-type *Nipponbare* and transgenic plants were inoculated with GD08-T13 at the three- to four-leaf stage. The values are means ± SDs of three biological replicates and the asterisks represent significant differences relative to *Nipponbare* plants at **P < 0.01 or *P < 0.05 by *t*-tests. The transcript level of *Nipponbare* plants was set to “1” at each time point

To further confirm the functions of *OsWRKY67* in rice disease resistance, we produced *OsWRKY67-*silenced (WRKY67-RNAi) plants of *Nipponbare* using an RNAi vector. No remarkable differences were observed between the transgenic and wild-type *Nipponbare* plants. Three independent transgenic lines (RNAi-9, RNAi-11, RNAi-15) in which *OsWRKY67* was most severely suppressed were used for disease resistance evaluation (Fig. 4a). Punch inoculation of plants revealed that the lesions on WRKY67-RNAi plants were approximately 50% larger than on *Nipponbare* plants (Fig. 4b, c) and the WRKY67-RNAi plants had more spores than *Nipponbare* plants, suggesting that the rate of fungal growth was faster in the WRKY67-RNAi plants relative to *Nipponbare* plants (Fig. 5d). Moreover, the WRKY67-RNAi plants also showed increased susceptibility to *M. oryzae* at the heading stage, with the infected main axis length ranging from 63.2 to 90% (82.38%), versus 47.5% for wild-type *Nipponbare* (Fig. 4e, f). To evaluate the resistance of the WRKY67-RNAi plants to bacterial blight, the leaves of the WRKY67-RNAi and *Nipponbare* plants were inoculated with isolate Chinese *Xoo* race 4 at the booting stage. The lesion length in WRKY67-RNAi plants was longer than that in the control plants, and the number of bacteria on RNAi plants was also much more than that on *Nipponbare* plants at 12 and 16 days after infection (Additional file 7: Figure S6). These results together indicate the positive role of *OsWRKY67* in regulating disease resistance in rice.

### Differential expression of defense-related genes in *OsWRKY67* overexpressing plants

To learn about the regulatory mechanisms of *OsWRKY67* in disease resistance in rice, global gene expression profiling was performed in OX-WRKY67 and wild-type *Nipponbare* plants by high through-put sequencing. In total, 345 genes were differentially expressed between OX-WRKY67 and *Nipponbare* plants, including 200 up-regulated genes and 145 down-regulated genes [**|**log_2_ Ratio**|** ≥ 1 and the False Discovery Rate (FDR) ≤ 0.01; Additional file 8: Table S2]. The results of the RNA-seq analysis were verified by real-time PCR for nine up-regulated genes and three down-regulated genes. As shown in Additional file 9: Figure S7, the transcription levels s of two *PR4* genes, four chitinases and three peroxidases were markedly induced whereas the three WRKY genes were all suppressed in OX-WRKY plants relative to *Nipponbare* plants. These results are consistent with the sequencing data, indicating the reliability of results from the RNA-seq analysis (Additional file 10: Figure S8). In addition, the transcript levels of these twelve genes were also analyzed in OX-WRKY and *Nipponbare* plants after pathogen infection. They all showed a similar differential expression pattern after blast inoculation (Additional file 9: Figure S7), further confirming that these genes were under the control of *OsWRKY67.*

Among the differentially expressed genes, 59 have been previously demonstrated to be involved or implicated in plant defense response (Additional file 8: Table S2; genes in red). The up-regulated genes in OX-WRKY67 plants included ten *PR* genes, five chitinase genes, three peroxidase genes and two phenylalanine ammonia-lyases (*PAL*). Overexpression of *OsWRKY67* also activated the transcription of other defense-related genes, such as laccases, cellulose synthases, dirigents and protease inhibitors (Additional file 8: Table S2). In contrast, the transcription of three *WRKY* genes (*WRKY60*, *WRKY7* and *WRKY74*) was significantly suppressed in OX-WRKY67 plants compared with those in wild-type plants. Moreover, several genes which are speculated to be associated with defense-related signal transduction, including protein kinases and other transcription factors (MYB, NAC and zinc finger), were also down-regulated in OX-WRKY67 plants (Additional file 8: Table S2).

Gene ontology (GO) analysis of these differentially expressed genes (DEGs) revealed 15 categories of enriched genes. Particularly, “cell wall organization”, “cellulose biosynthesis”, “chitin metabolism” and “response to biotic stimulus” were remarkably enriched for the up-regulated genes, while different “transport” processes were enriched for the down-regulated genes (Additional file 11: Table S3). Furthermore, pathway analysis indicated that these DEGs were mainly involved in ribosomes, biosynthesis of secondary metabolites, metabolic pathway and plant-pathogen interactions (Additional file 12: Table S4).

### *OsWRKY67* acts in the salicylic acid dependent pathway

From our observation of the highly induced transcription of PR genes and other defense-related genes, we deduced that *OsWRKY67*’s functional role in disease resistance may, at least, partially dependent on the SA signaling pathway. To validate this inference, we first analyzed the expression levels of several well-known defense-related genes in the OX-WRKY67, WRKY67-RNAi and wild-type *Nipponbare* plants. Isochorismate synthase 1 (*ICS1*) and phenylalanine ammonia-lyase 1 (*PAL1*) are two SA synthesis genes, while phytoalexin-deficient 4 (*PAD4*), *Arabidopsis* NPR1 homolog 1 (*NHI*), *PR1a* and *PR10* are four genes that function in the SA signaling pathway [17, 27]. Our results showed that *ICS1*, *PAD4*, *PAL1*, *NH1*, *PR1a* and *PR10* were significantly induced in OX-WRKY67 plants compared with those in *Nipponbare* plants after inoculation with *M. oryzae* (Fig. 5a). Among these six genes, the expression of *PAL1*, *PR1a* and *PR10* were remarkably higher in transgenic plants than in *Nipponbare* plants both before and after blast inoculation, while the higher transcript levels of *ICS1*, *PAD4* and *NH1* were only identified after pathogen inoculation (Fig. 5a). In contrast, the transcription levels of *PAD4*, *PAL1*, *NH1*, *PR1a* and *PR10* were remarkably lower in WRKY67-RNAi plants compared to *Nipponbare* plants after pathogen inoculation (Fig. 5b). No obvious difference was detected for *ICS1* between WRKY67-RNAi and *Nipponbare* plants (Fig. 5b). Moreover, the transcript levels of these six genes were also analyzed in the transgenic plants and *Nipponbare* plants at the booting stage after *Xoo* infection. Consistent with the results from the blast inoculation experiments, the transcription of these genes was induced in OX-WRKY67 plants and down-regulated in WRKY67-RNAi plants relative to the *Nipponbare* plants (Additional file 13: Figure S9).

**Fig. 6:**
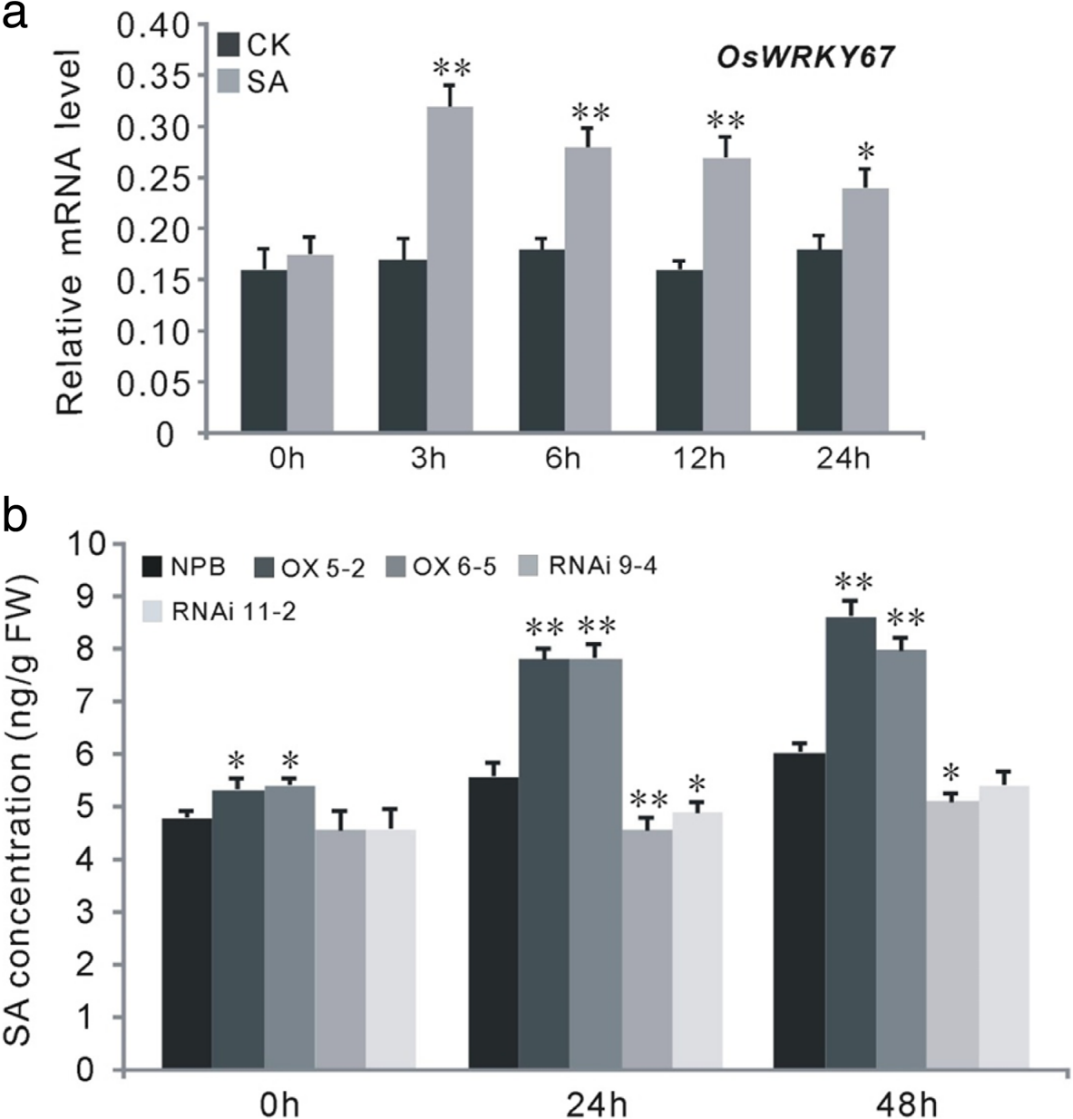
*OsWRKY67* confers disease resistance in a SA-dependent manner. The values are means ± SDs of three biological replicates and the asterisks represent significant differences relative to the water treatment (0 h) or *Nipponbare* plants at **P < 0.01 and *P < 0.05 by *t*-test. **a** The expression of *OsWRKY67* was activated by exogenous SA. CK = water treatment. **b** Transcriptionally modulating *OsWRKY67* influenced the accumulation of SA

Next, we analyzed the transcription of *OsWRKY67* after treating the wild-type *Nipponbare* plants with exogenous SA. The SA treatment significantly induced expression of *OsWRKY67* at all measured time points after treatment (Fig. 6a). We also measured the contents of endogenous SA in the seedlings of OX-WRKY67, WRKY67-RNAi and *Nipponbare* after inoculation with blast isolate GD08-T13 at the three- to four-leaf stage (Fig. 6b). The concentration of SA was elevated due to blast infection in both OX-WRKY67 and *Nipponbare* plants, but the SA level was remarkably higher in OX-WRKY67 plants compared with wild-type *Nipponbare* plants before and after blast infection (Fig. 6b). On the contrary, the endogenous SA content was significantly lower (*P* < 0.05) in the WRKY67-RNAi plants compared to *Nipponbare* plants after pathogen attack (Fig. 6b). These observations together suggest that there is a close relationship between the *OsWRKY67*-mediated defense response and the SA signaling pathway.

### *OsWRKY67* directly regulates the expression of *PR1a* and *PR10*

**Fig. 7:**
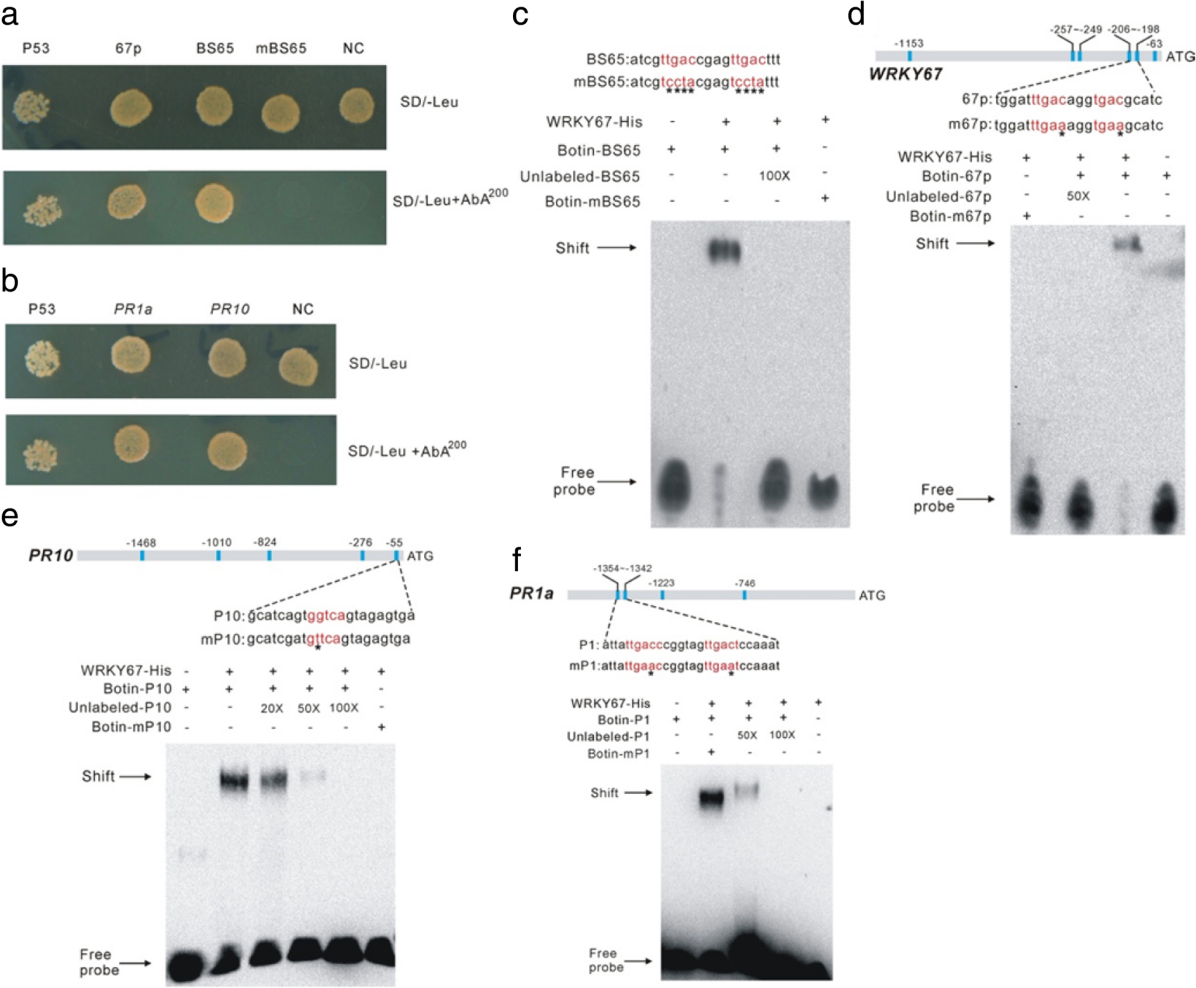
*OsWRKY67* binds specifically to the W-box elements. **a** Yeast one-hybrid assays indicated that *OsWRKY67* can bind directly to the sequences of W-box (BS65) and its own promoter (67p). P53 is the positive control. NC is the negative control which did not contain the W-box and mBS65 is the mutated probe of BS65 with TGAC sequence changed to CCTA. The detailed sequences of BS65 and mBS65 are shown in section c. **b** Yeast one-hybrid assays indicated that *OsWRKY67* can bind directly to the promoters of *PR1a* and *PR10* through the W-box motif. NC is the negative control from the *PR10* promoter which did not contain the W-box motif. **c** The EMSA experiment showed the binding of OsWRKY67 protein to the sequences of the W-box. An excess of unlabeled-BS65 probe was added as competitors. The negative control was conducted by inoculating the Biotin-labeled BS65 probe without the OsWRKY67-His protein. **d** The EMSA experiment showed the binding of OsWRKY67 protein to its own promoter through the W-box element. The oligonucleotides 67p and m67p were used as the probes. An excess of unlabeled-67p probe was added as competitors. **e** The EMSA experiment showed the binding of OsWRKY67 protein to the promoter of *PR10*. The oligonucleotides P10 and mP10 were used as the probes. An excess of unlabeled-P10 probe was added as competitors. **f** The EMSA experiment showed the binding of OsWRKY67 protein to the promoter of *PR1a*. The oligonucleotides P1 and mP1 were used as the probes. An excess of unlabeled-P1 probe was added as competitors

The WRKY transcription factors regulate the expression of downstream genes by binding to the promoters of these genes via the W-box elements. To determine the DNA-binding activity of OsWRKY67, we performed a yeast one-hybrid assay using DNA fragments (BS65 and mBS65) according to previous reports [24, 29]. The assay showed that OsWRKY67 strongly bonded to the probe of BS65, whereas OsWRKY67 did not recognize the mutant probe mBS65, implying the specificity in DNA-binding for OsWRKY67 (Fig. 7a). Interestingly, we also identified multiple W-box sequences in the promoters of *OsWRKY67*, *PR1a*, *PR10*, *ICS1* and *NH1,* and the yeast one-hybrid assays indicated that *OsWRKY67* could bind to its own promoter as well as the promoters of *PR1a* and *PR10* through the interaction with the W-boxes (Fig. 7a, b). The direct interactions between *OsWRKY67* and the promoters of *ICS1* and *NH1* were not identified.

To further validate the binding specificity of OsWRKY67 to these W-box elements, we performed electrophoretic mobility-shift assays (EMSA) with an OsWRKY67-histone (His) fusion protein. As shown in Fig. 7c, d-f, no binding signal was detected for the control (non-fusion protein, lane 1). The fusion protein could bind to the BS65, 67p, P1 and P10 probes, but could not bind to the mBS65, m67p, mP1 and mP10 probes, in which the TGAC motifs were mutated. Moreover, the specific binding of the fusion protein to BS65, 67p, P1 and P10 was eliminated by addition of unlabeled competitors (Fig. 7c, d-f). Taken together, these results indicated that OsWRKY67 directly binds to the W-box elements in the promoters of *PR1a* and *PR10* to activate its expression. Moreover, our results also demonstrated that *OsWRKY67* could bind to its own promoter, implying that the expression of *OsWRKY67* could be self-regulated.

## Discussion

As one of the largest families of transcriptional regulators specifically expressed in plants, the pivotal roles of WRKY proteins in regulating disease resistance and abiotic stress response have been well-documented in many plant species [7–13]. In rice, more than 100 WRKY proteins have been identified and 11 of them have been demonstrated to be associated with defense responses [15–22]. However, only *OsWRKY45* has been reported on regarding its regulatory role in panicle blast [28]. Because panicle blast is more destructive to grain yield than leaf blast, the gaps in knowledge of the roles of other members of this family in panicle blast resistance need to be filled. Here, the functions of *OsWRKY67* in leaf blast, panicle blast and bacterial blight have been demonstrated. *OsWRKY67* was significantly induced after infection with leaf blast and panicle blast. The plants constitutively expressing *OsWRKY67* were more resistant to leaf blast and panicle blast as manifested by smaller lesion size and lower fungal growth when compared with wild-type *Nipponbare* plants. *OsWRKY67-*silenced plants, on the other hand, exhibited increased susceptibility to blast disease as manifested by larger lesion size and greater fungal growth. Furthermore, *OsWRKY67*-overexpressing plants also exhibited enhanced resistance to *Xoo* as indicated by their smaller lesions and lower bacterial growth rates. Altogether, these results together suggest a positive role of *OsWRKY67* in rice-*M. oryzae* and rice-*Xoo* interactions.

Furthermore, we have demonstrated that *OsWRKY67*-overexpressing plants exhibited a dwarfed phenotype and produced lower numbers of tillers relative to the wild-type *Nipponbare* plants, indicating that *OsWRKY67* also functions in regulating plant growth and development. However, there were no significant phenotypic differences between WRKY67-RNAi and *Nipponbare* plants. Interestingly, we also identified that the transcription of *OsWRKY67* was modulated by cold, high salinity, dehydration, oxidative and abscisic acid stresses, and *OsWRKY67* overexpressing plants showed increased tolerance to dehydration stress whereas overexpression of *OsWRKY67* led to increased sensitivity to dehydration stress (unpublished data). Therefore, *OsWRKY67* may have multiple roles in regulating rice development and various stress responses.

WRKY proteins regulate plant defense response by distinct signaling pathways where many WRKY proteins positively regulate plant defense responses while others negatively regulate disease resistance [15–22]. Most of the defense pathways mediated by WRKY proteins were either SA-dependent or jasmonic acid (JA)-dependent. For example, rice *WRKY45* functions in benzothiadiazole-inducible blast resistance by modulating the SA signaling pathway [18], while *WRKY11* and *WRKY17* negatively regulate the basal resistance of *Arabidopsis* in a JA-dependent pathway [8]. In the present study, our data have demonstrated the positive role of *OsWRKY67* in plant disease resistance and the resistance is dependent on the SA signaling pathway. First, many genes that are involved in SA synthesis or SA-conferred defense response were significantly up-regulated in *OsWRKY67* overexpressing plants relative to wild-type *Nipponbare* plants. Second, the transcription of *OsWRKY67* was markedly induced by exogenous SA. Third, the endogenous level of SA was remarkably higher in *OsWRKY67*-overexpressing plants whereas the SA content was lower in *OsWRKY67*-silenced plants compared with *Nipponbare* plants. Fourth, *OsWRKY67* directly induced the expression of *PR1a* and *PR10* by binding to their promoters via W-box elements. These data strongly suggest that *OsWRKY67* positively regulates rice disease resistance in a SA-dependent pathway. In Arabidopsis, *NPR1* has been validated to play a major role in transducing the SA signal by activation of downstream defense-related genes [30]. In this study, we identified that *NH1*, a rice homolog of *NPR1*, was significantly induced in *OsWRKY67* overexpressing plants relative to *Nipponbare* plants both before and after pathogen infection. This suggests that *OsWRKY67*-mediated disease resistance may be associated with a *NH1*-dependent SA signaling pathway. Further research will be needed to verify this inference.

In addition to its function as a positive regulator, *OsWRKY67* is also a negative regulator of gene expression. Among the genes down-regulated in *OsWRKY67*-overexpressing plants, three WRKY (*WRKY60*, *WRKY7* and *WRKY74*) genes were identified, suggesting that *OsWRKY67* may act upstream of these genes. In Arabidopsis, *WRKY7* has been demonstrated to negatively regulate plant disease resistance [31]. In this study, the down-regulated expression of *WRKY7* in *OsWRKY67*-overexpressing plants further indicated the positive role of *OsWRKY67* in plant defense response.

Many studies have shown that WRKY proteins can directly regulate the transcription of their target genes by binding to the W-boxes in the promoter of these genes. Since the promoters of *WRKY60*, *WRKY7* and *WRKY74* contains many W-boxes (data not shown), their expression may be regulated directly by *OsWRKY67*. Alternatively, these WRKY genes can also be regulated by other WRKY genes that function downstream of *OsWRKY67*. Notably, in this study, we also observed several W-boxes in the promoter of *OsWRKY67*, and our results indicated that *OsWRKY67* could bind to its own promoter and therefore, could auto-regulate itself. Moreover, except for the possibility of self-regulation, the expression of *OsWRKY67* is probably modulated directly by other upstream WRKY proteins. These results exhibit an intricate network of autoregulation and cross-regulation in the *OsWRKY67*-mediated disease resistance pathways in rice. However, the cross-regulation between distinct WRKY proteins during plant defense response seems to be a general characteristic of the WRKY family. For instance, *AtWRKY18* contains many W-box elements in its promoter and these elements function as negative regulators [32], whereas *AtWRKY6* negatively regulates not only its own expression but also the transcription of *WRKY42* [33]. Together, these observations indicate that WRKY proteins form a highly interconnected network in plants, and this network modulates distinct WRKY gene expression during the defense response and thereby, fine-tune the plant defense response.

Based on the findings of the potential cross-regulation network of WRKY proteins in plant defense responses as well as the important role of *OsWRKY45* in regulating rice panicle blast resistance [28, 34], we deduced that if the *OsWRKY67*-mediated disease resistance is involved in modulating the expression of *OsWRKY45* in rice. To verify this inference, the transcription levels of *OsWRKY45* were analyzed in the wild-type *Nipponbare* and *OsWRKY67*-overexpressing and silenced plants at normal conditions. Simultaneously, the expression levels of *OsWRKY13*, *OsWRKY30* and *OsWRKY31*, which are involved in leaf blast resistance [17, 20, 35], were also analyzed in these plants. Unfortunately, no significant differences were observed in the expression of these WRKY genes between wild-type *Nipponbare* and the transgenic plants (Additional file 14: Figure S10), indicating that *OsWRKY67*-mediated disease resistance is not involved in regulating the expressions of *OsWRKY45*, *OsWRKY13*, *OsWRKY30* and *OsWRKY31* in rice.

## Conclusion

In the present study, we have demonstrated that *OsWRKY67* positively regulates rice resistance to leaf blast, panicle blast and bacterial blight diseases. *OsWRKY67* confers disease resistance by the induction of a series of defense–related genes and the activation of the SA signaling pathway with *PR1a* and *PR10* as the direct targets of *OsWRKY67*. Moreover, *OsWRKY67* can also bind to its own promoter via the W-box element, suggesting self-regulation in *OsWRKY67*-mediated biological processes. However, we still do not know whether there are other TFs, including WRKYs, acting upstream of *OsWRKY67* and how *OsWRKY67* may coordinate with the potential WRKY genes to contribute to disease resistance in rice. Further study will be required to address these issues. Resistance to the two major diseases attributable to *OsWRKY67* makes it a promising target of molecular breeding for crop improvement in rice.

## Methods

### Plant growth and hormone treatments

The japonica rice (*Oryza sativa L.*) *Nipponbare* and a blast-resistant line BC10 were used in this study. Collection of the two rice varieties was complied with the institutional and national guidelines in China. Surface-sterilized rice seeds were planted onto 1/2 MS medium for germination and growth. Two-weeks later, the seedlings were transplanted into bucket and kept in the net-house of Rice Research Institute, Guangdong Academy of Agricultural Sciences, in Guangzhou (23^°^06′N, 113^°^15′E). Hormone treatments were performed using the same method according to our previous report [27]. The leaves of seedlings at the three- to four-leaf stage were gently watered with a SA solution at a concentration of 100 μM. Control treatment seedlings were sprayed with water.

### Plasmid construction and transformation

To build the overexpressing plasmid, the coding sequence of *OsWRKY67* amplified from BC10 using OsWRKY67-OE-F/R (Additional file 15) was sub-cloned into the pOx overexpressing vector [36]. A 210-bp partial sequence of *OsWRKY67* was amplified by RT-PCR using primers OsWRKY67-RNAi-F/R (Additional file 15), and was then inserted into the pRNAi-Ubi vector [27] to generate the *OsWRKY67* RNAi plasmid. For promoter analysis, ~ 2.4-kb fragments upstream of *OsWRKY67* amplified using primers OsWRKY67-GUS-F/R (Additional file 15) was sub-cloned into the pCAMBIA1381Z vector. All the plasmid constructs were verified by sequencing and then were transferred into *Agrobacterium tumefaciens* EHA105 for transformation of *Nipponbare* plants via an Agrobacterium-mediated genetic transformation approach.

### Evaluation of disease resistance

Isolate GD08-T13 of *Magnaporthe oryzae* was used for rice blast inoculation and the Chinese *Xanthomonas oryzae pv. Oryzae* (*Xoo*) race 4 isolate was used for *Xoo* inoculation. Evaluation of blast and *Xoo* resistance was conducted using the same method described in our previous reports [27, 37]. For spray inoculation, seedlings at the three- to four- leaf stage were sprayed with a spore suspension (1 × 10^6^ spores/mL) containing 0.05% Tween-20. The disease was assessed 7 days after inoculation by counting the number of lesions per leaf. Six-week-old plants were punch inoculated as described previously [38, 39]. Briefly, 5 μL of a spore suspension (5 × 10^5^ spores/mL) containing 0.05% Tween-20 was added to the press-injured spots on fully expanded rice leaves. The inoculated spots were wrapped with transparent scotch tape, and leaves were photographed 12 days after inoculation to measure lesion size by the ImageJ program (http://imagej.nih.gov/ij/). For determination of *in planta* sporulation after punch inoculation, leaf strips containing a lesion spot were excised and submerged in 100 μL of distilled water in a 1.5 mL microcentrifuge tube. After the suspension was vigorously mixed, spores were counted with a microscope [39].

For panicle blast inoculation, the cotton-wrapping inoculation method was used as described previously [27]. The upper-middle part of a panicle was wrapped with sterile cotton within 2 to 3 days after heading. Two milliliters of a suspension of 1 × 10^6^ spores/ml of *M. oryzae* GD08-T13 was injected into the cotton after which the cotton was wrapped with foil. The inoculated plants were sprayed with water for 3 min every 3 h to maintain the humidity. Evaluation of panicle blast resistance was conducted at 3 weeks after inoculation by measuring the percent of infected main axis length.

To better distinguish the resistance phenotypes between silenced plants and control plants (*Nipponbare*), lower concentrations of spore suspension [about 0.5 × 10^6^ spores / ml for panicle blast and 2.5 × 10^5^ spores / ml for leaf blast (punch method)] were used.

### GUS staining analysis

Analysis of GUS activity in different rice tissues was conducted as described previously [27].

### Subcellular localization of OsWRKY67 protein

The CDS sequence of *OsWRKY67* amplified using primers OsWRKY67-GFP-F/R (Additional file 15) was inserted into the 35S-GFP vector to produce the OsWRKY67-GFP fusion protein. Next, the fusion plasmid and the empty 35S-GFP plasmid were transferred into onion epidermal cells or rice stem protoplasts as described previously [37, 40]. Laser confocal microscopy (Zeiss LSM710, Germany) was used to detect the GFP fluorescence after 24 h of incubation at 25 °C.

### Yeast one-hybrid assay and EMSA

The yeast one-hybrid assays and EMSA experiments were performed using the same method according to our previous report [27]. For yeast one-hybrid assays, about 350 bp fragments containing at least three W-boxes were amplified from the promoters of *OsWRKY67*, *PR1a* and *PR10*. Then, the cloned sequences were inserted into the pAbAi vector to generate the bait protein. A negative control (NC) was included by using a 100 bp sequence without a W-box from the promoter of *OsWRKY67*. Primers used for the EMSA experiments were labeled by the EMSA Probe Biotin Labeling Kit (Beyotime, China). The un-labeled primers were employed as the competitors.

### Real-time PCR analysis

Total RNA was extracted from different rice tissues using Eastep® Super Total RNA Extraction Kit (Promega Biotech Co., Ltd., USA). Inverse transcription and real-time PCR were conducted using the same method as described previously [27]. Gene-specific primers used in this study are shown in Additional file 15.

### Quantification of endogenous SA

The SA was extracted and measured using the same protocol in our previous report [37]. The optimized MS/MS conditions used for quantification of endogenous SA are listed in Additional file 16: Table S6.

### RNA-seq analysis

Two-week old leaves of wild-type *Nipponbare* and *OsWRKY67* overexpressing plants were collected for RNA-seq analysis. Solexa sequencing was conducted by the ANNOROAD Gene Technology Co. LTD (Beijing, China). The raw data was submitted to the Sequence Read Archive of the National Center of Biotechnology Information (NCBI) (accession number: SRP096216). For data analysis, clean reads were obtained by removing low quality tags, adaptor sequences and other contaminants. Next, the clean reads were all mapped to the *Oryza sativa* genome (MSU7) using the bowtie2 program(v2.2.3) [41] and tophat2 program (v2.0.12) [42] with default parameter values. Cufflinks program (v2.2.1) [43] was used for estimation of the FPKM (Fragments Per Kilobase per Million mapped reads) values to obtain the gene expression level. The program edgeR (v3.8.6) [44] was applied to detect differentially expressed genes with an FDR ≤ 0.05 and a relative change threshold of two-fold. An online server named PlantGSEA [45] was used for enrichment analysis of biological processes with the Plant Ontology, Gene Ontology and KEGG pathway databases.

## Additional files

Additional file 1: **Table S1.** The microarray data of *OsWRKY67* after leaf blast and panicle blast infection. (XLSX 9 kb)

Additional file 2: **Figure S1.** Subcellular localization of OsWRKY67. Bar = 2 μm. **a** Laser confocal microscopy images deriving from GFP of onion epidermal cells transiently expressing GFP or GFP-OsWRKY67 fusion protein. Arrows indicate the nucleus. **b** Laser confocal microscopy images deriving from GFP of rice protoplasts transiently expressing GFP or GFP-OsWRKY67 fusion protein. (JPG 1354 kb)

Additional file 3: **Figure S2.** Phenotypes of wild-type *Nipponbare* (NPB) and *OsWRKY67*-overexpressing (OX-WRKY67) plants at normal conditions. The values are means ± SDs of twenty biological replicates and the asterisks represent significant differences relative to *Nipponbare* plants at **P < 0.01 by *t*-test. **a** Phenotypes of *Nipponbare* and OX-WRKY67 transgenic lines OX 5–2 and OX 6–5 at the heading stage. **b** Plant heights of *Nipponbare* and OX-WRKY67 transgenic lines OX 5–2 and OX 6–5. **c** Tiller numbers of *Nipponbare* and OX-WRKY67 transgenic lines OX 5–2 and OX 6–5. (JPG 1090 kb)

Additional file 4: **Figure S3.** Southern blot analysis of the copy numbers of transgene. (JPG 439 kb)

Additional file 5: **Figure S4.** Phenotypes of OX-WRKY67 and *Nipponbare* plants infected with leaf blast using spraying method. a The OX-WRKY67 plants exhibited enhanced leaf blast resistance after inoculation with GD08-T13 using spraying method. b The lesion numbers per leaf of *Nipponbare* and OX-WRKY67 plants after inoculation with GD08-T13. The values are means ± SDs of twelve biological replicates. The asterisks represent significant differences relative to *Nipponbare* plants (*t*-test, **P < 0.01). (JPG 348 kb)

Additional file 6: **Figure S5.** Phenotypes of the OX-WRKY67 and *Nipponbare* plants infected with bacterial blight. The values are means ± SDs of twenty biological replicates and the asterisks represent significant differences relative to *Nipponbare* plants at **P < 0.01 by *t*-test. **a** Disease phenotypes of OX-WRKY67 and *Nipponbare* plants after *Xoo* inoculation. **b** Lesion lengths in OX-WRKY67 and *Nipponbare* plants after *Xoo* inoculation. **c** Growth rates of *Xoo* race 4 in the leaves of OX-WRKY67 and *Nipponbare* plants. Bacterial populations were determined from three leaves 12 or 16 days after inoculation by counting colony-forming units (cfu). Similar results were obtained in two independent experiments. (JPG 665 kb)

Additional file 7: **Figure S6.** Phenotypes of *Nipponbare* and *OsWRKY67*-silenced (WRKY67-RNAi) plants infected with bacterial blight. The values are means ± SDs of twenty biological replicates and the asterisks represent significant differences relative to *Nipponbare* plants at **P < 0.01 and *P < 0.05 by *t*-tests. **a** Disease phenotypes of WRKY67-RNAi and *Nipponbare* plants after *Xoo* inoculation. **b** Lesion lengths in WRKY67-RNAi and *Nipponbare* plants after *Xoo* inoculation. **c** Growth rates of *Xoo* race 4 in the leaves of WRKY67-RNAi and *Nipponbare* plants. Bacterial populations were determined from three leaves 12 or 16 days after inoculation by counting colony-forming units (cfu). Similar results were obtained in two independent experiments. (JPG 711 kb)

Additional file 8: **Table S2.** Differentially expressed genes in *OsWRKY67* overexpressing plants by RNA-seq analysis. (XLSX 26 kb)

Additional file 9: **Figure S7.** Overexpression of *OsWRKY67* influences the expression of defense-related genes. The values are means ± SDs of three biological replicates and asterisks represent significant differences relative to *Nipponbare* plants before blast inoculation (0 h) or at 12, 24 and 48 h after inoculation with *M. oryzae* (*t*-test, **P < 0.01 and *P < 0.05). **a** Nine defense-related genes were over-presented in OX-WRKY67 plants. **b** Three *WRKY* genes were suppressed in OX-WRKY67 plants. (JPG 918 kb)

Additional file 10: **Figure S8.** The results of qRT-PCR showed an excellent concordance with the sequencing data. (JPG 214 kb)

Additional file 11: **Table S3.** Gene ontology analysis of up- and down-regulated genes in *OsWRKY67-*overexpressing plants detected by RNA-seq analysis. (XLSX 12 kb)

Additional file 12: **Table S4.** A statistic pathway enrichment analysis of differentially expressed genes between *OsWRKY67*-overexpressing plants and *Nipponbare* plants. (XLSX 11 kb)

Additional file 13: **Figure S9.** *OsWRKY67* regulates the expression of a set of defense-related genes at 12 h and 36 h after bacterial blight infection. The wild-type *Nipponbare* and transgenic plants were inoculated with *Xoo race 4* at the booting stage. The values are means ± SDs of three biological replicates and the asterisks represent significant differences relative to *Nipponbare* plants at **P < 0.01 or *P < 0.05 by *t*-tests. The expression of *Nipponbare* plants was set to “1” at each time point. **a** Relative expression levels of defense-related genes in *Nipponbare* and OX-WRKY67 plants. **b** Relative expression levels of defense-related genes in *Nipponbare* and WRKY67-RNAi plants. (JPG 1135 kb)

Additional file 14: **Figure S10.** Relative expression levels of four WRKY genes in wild-type *Nipponbare* and *OsWRKY67* transgenic plants. The values are means ± SDs of three biological replicates. (JPG 381 kb)

Additional file 15: **Table S5.** Primers used for quantitative RT-PCR and vector construction. (XLSX 10 kb)

Additional file 16: **Table S6.** Characteristic fragment ions of the SA standard and its optimized MS/MS condition. (XLSX 10 kb)

## Abbreviations

DEGs: Differentially expressed genes
FDR: False discovery rate
GFP: Green fluorescence protein
GO: Gene ontology
GUS: β-glucuronidase
ICS1: Isochorismate synthase 1
NC: Negative control
NCBI: National Center of Biotechnology Information
NHI: *Arabidopsis* NPR1 homolog 1
OX-WRKY67: *OsWRKY67* overexpressing
PAD4: Phytoalexin-deficient 4
PAL1: Phenylalanine ammonia-lyase 1
PR10: Pathogenesis-related [PR] protein 10
SA: Salicylic acid
TF: Transcription factor
WRKY67-RNAi: *OsWRKY67* silencing
*Xoo*: *Xanthomonas oryzae pv. Oryzae*
