# Supplementary figures and images for: OsWRKY67 positively regulates blast and bacteria blight resistance by direct activation of PR genes in rice
Source: BMC Plant Biol. 2018 Oct 26;18:257. doi: 10.1186/s12870-018-1479-y (PMC6204034; doi:10.1186/s12870-018-1479-y)

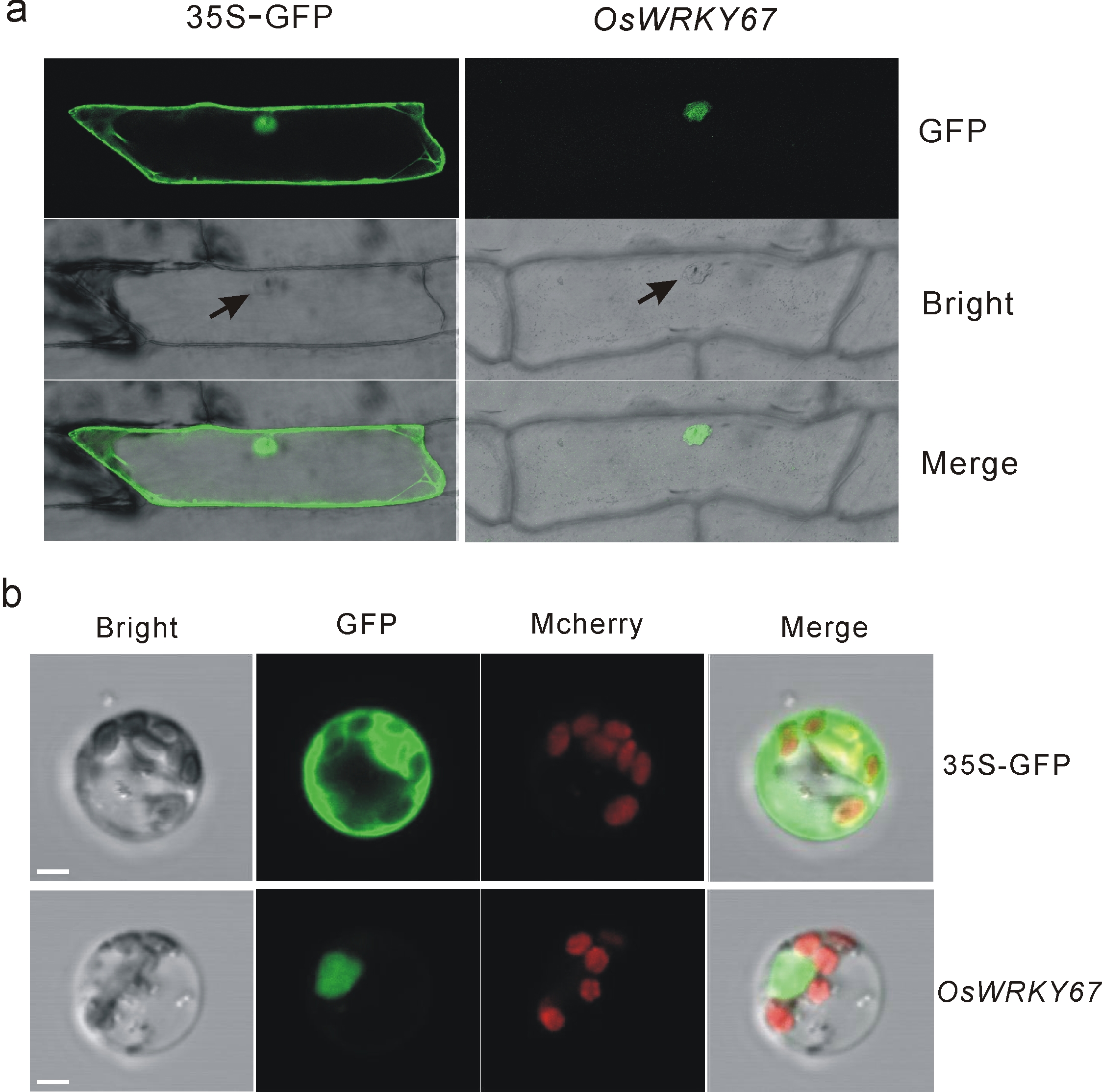

Supplement: Supplementary file 2 — Figure S1. Subcellular localization of OsWRKY67. Bar = 2 μm. a Laser confocal microscopy images deriving from GFP of onion epidermal cells transiently expressing GFP or GFP-OsWRKY67 fusion protein. Arrows indicate the nucleus. b Laser confocal microscopy images deriving from GFP of rice protoplasts transiently expressing GFP or GFP-OsWRKY67 fusion protein. (JPG 1354 kb) [file 12870_2018_1479_MOESM2_ESM.jpg]

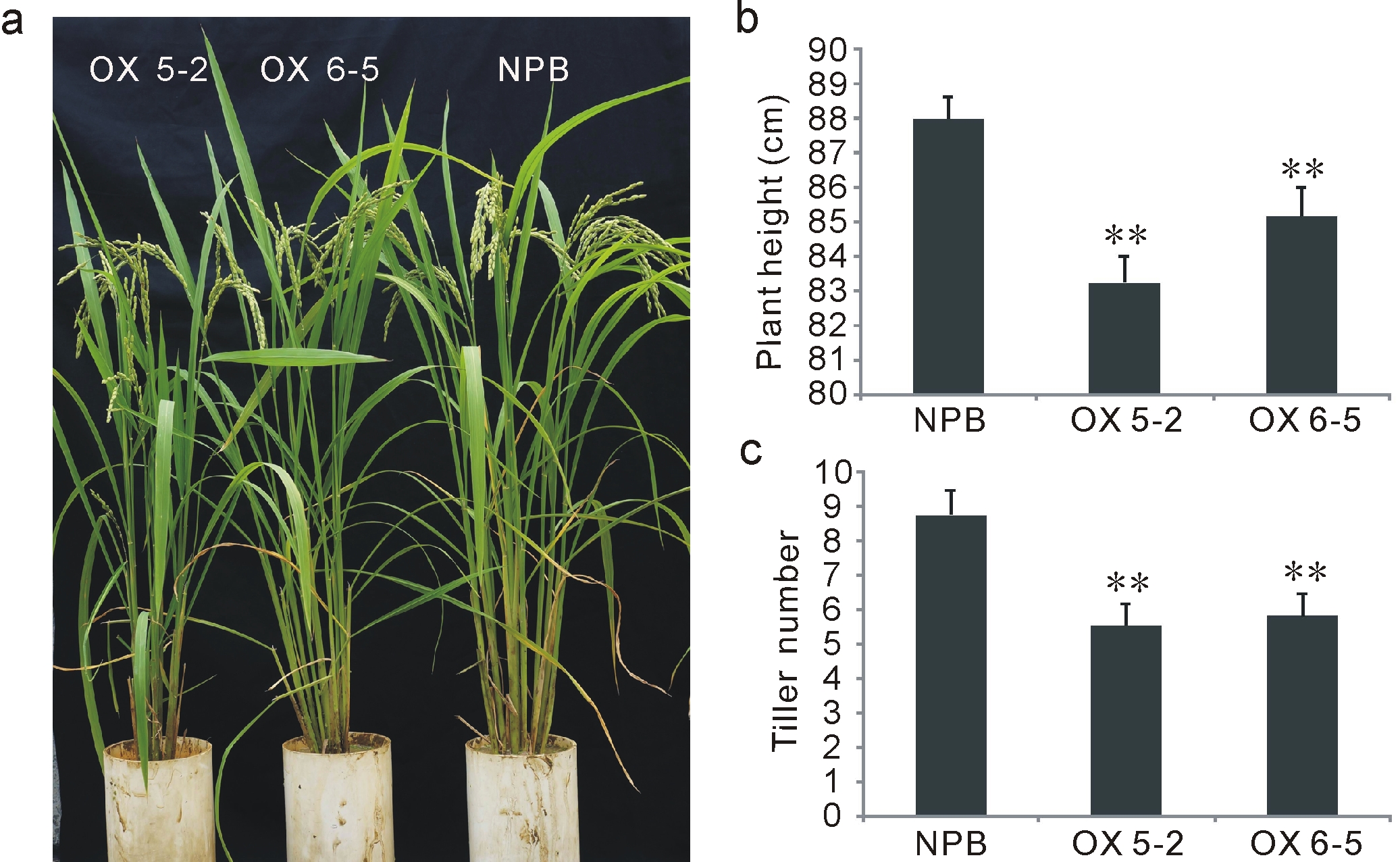

Supplement: Supplementary file 3 — Figure S2. Phenotypes of wild-type Nipponbare (NPB) and OsWRKY67-overexpressing (OX-WRKY67) plants at normal conditions. The values are means ± SDs of twenty biological replicates and the asterisks represent significant differences relative to Nipponbare plants at **P < 0.01 by t-test. a Phenotypes of Nipponbare and OX-WRKY67 transgenic lines OX 5–2 and OX 6–5 at the heading stage. b Plant heights of Nipponbare and OX-WRKY67 transgenic lines OX 5–2 and OX 6–5. c Tiller numbers of Nipponbare and OX-WRKY67 transgenic lines OX 5–2 and OX 6–5. (JPG 1090 kb) [file 12870_2018_1479_MOESM3_ESM.jpg]

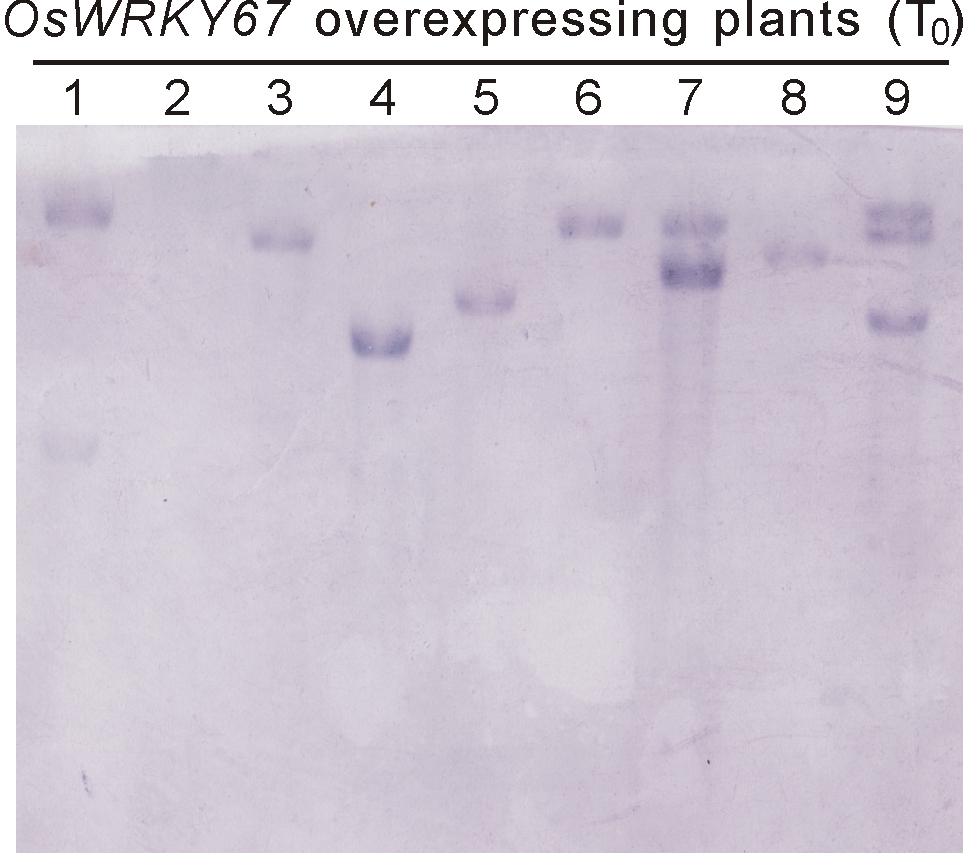

Supplement: Supplementary file 4 — Figure S3. Southern blot analysis of the copy numbers of transgene. (JPG 439 kb) [file 12870_2018_1479_MOESM4_ESM.jpg]

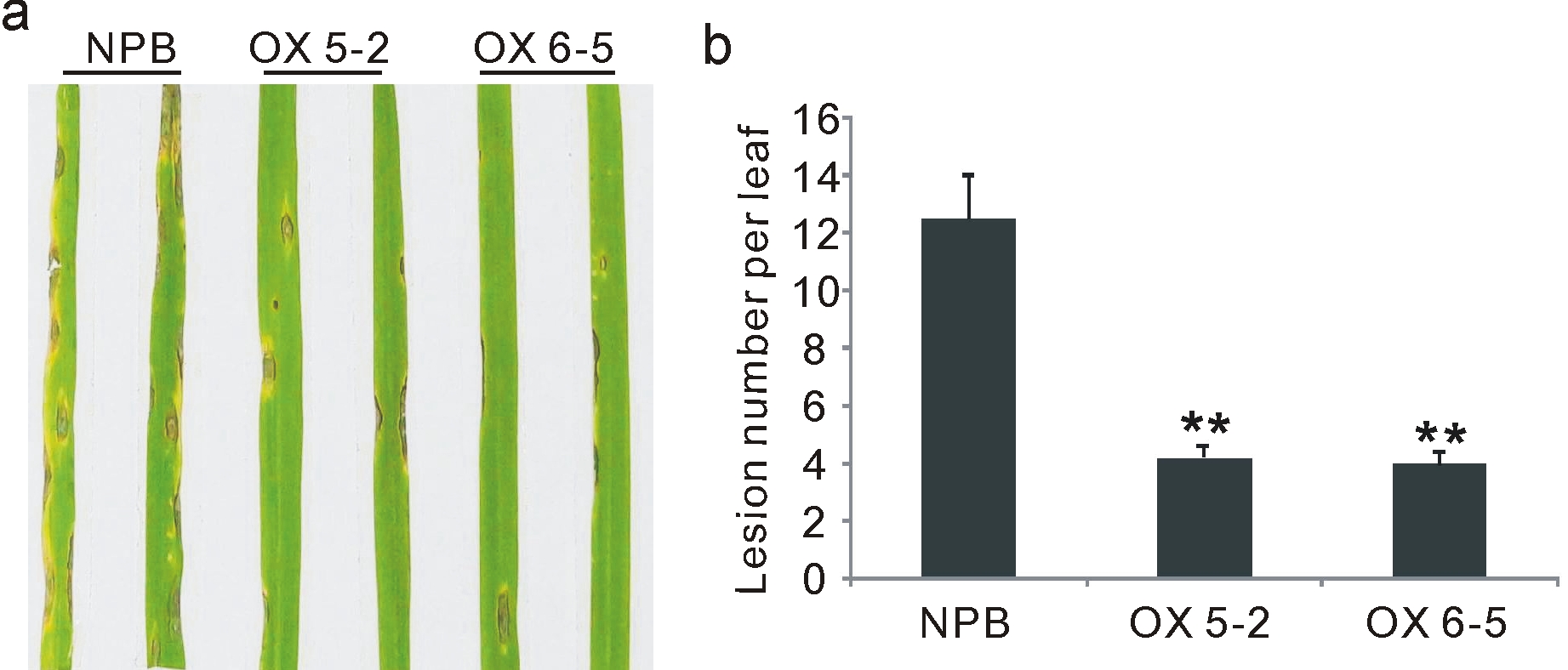

Supplement: Supplementary file 5 — Figure S4. Phenotypes of OX-WRKY67 and Nipponbare plants infected with leaf blast using spraying method. a The OX-WRKY67 plants exhibited enhanced leaf blast resistance after inoculation with GD08-T13 using spraying method. b The lesion numbers per leaf of Nipponbare and OX-WRKY67 plants after inoculation with GD08-T13. The values are means ± SDs of twelve biological replicates. The asterisks represent significant differences relative to Nipponbare plants (t-test, **P < 0.01). (JPG 348 kb) [file 12870_2018_1479_MOESM5_ESM.jpg]

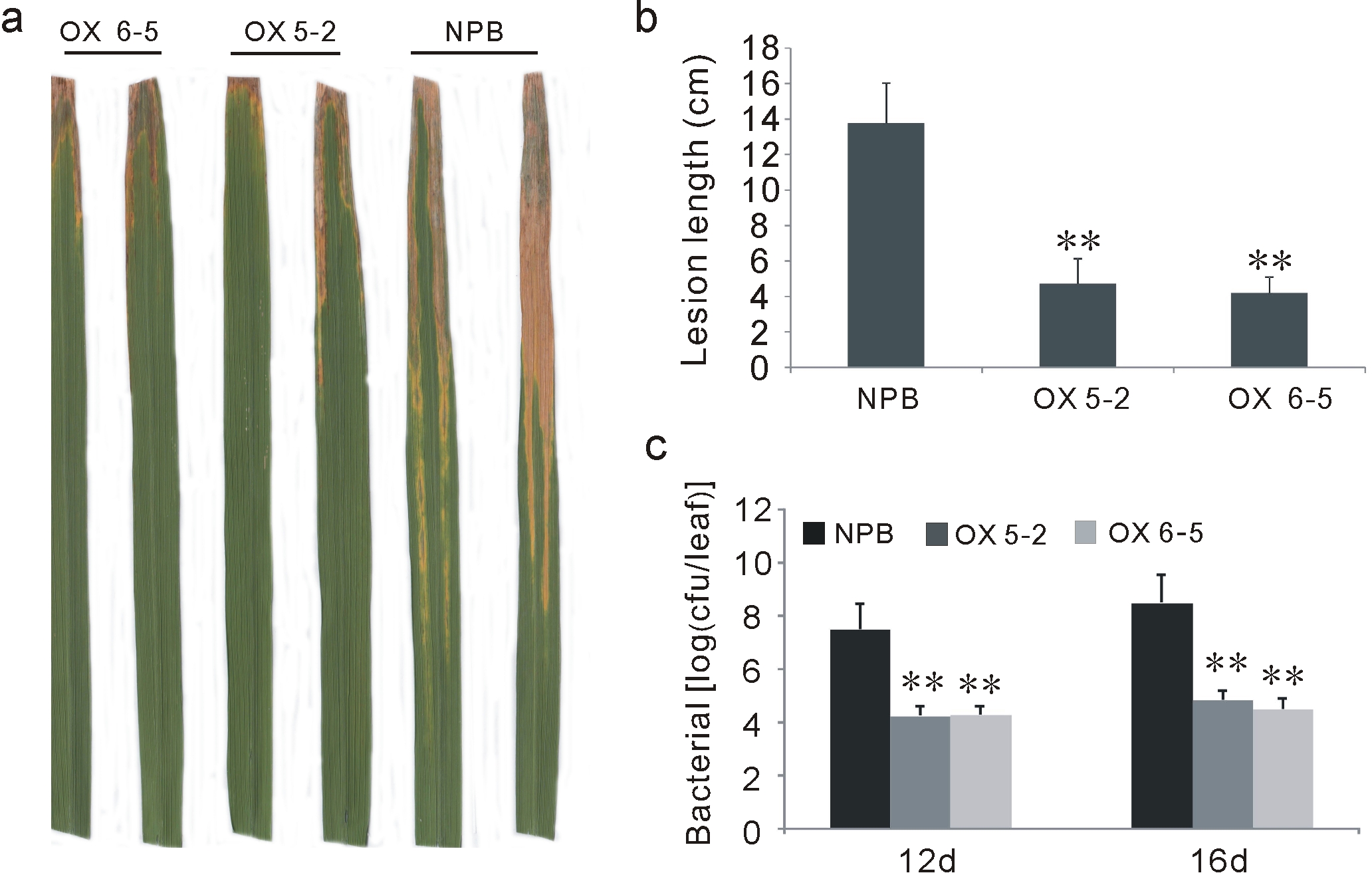

Supplement: Supplementary file 6 — Figure S5. Phenotypes of the OX-WRKY67 and Nipponbare plants infected with bacterial blight. The values are means ± SDs of twenty biological replicates and the asterisks represent significant differences relative to Nipponbare plants at **P < 0.01 by t-test. a Disease phenotypes of OX-WRKY67 and Nipponbare plants after Xoo inoculation. b Lesion lengths in OX-WRKY67 and Nipponbare plants after Xoo inoculation. c Growth rates of Xoo race 4 in the leaves of OX-WRKY67 and Nipponbare plants. Bacterial populations were determined from three leaves 12 or 16 days after inoculation by counting colony-forming units (cfu). Similar results were obtained in two independent experiments. (JPG 665 kb) [file 12870_2018_1479_MOESM6_ESM.jpg]

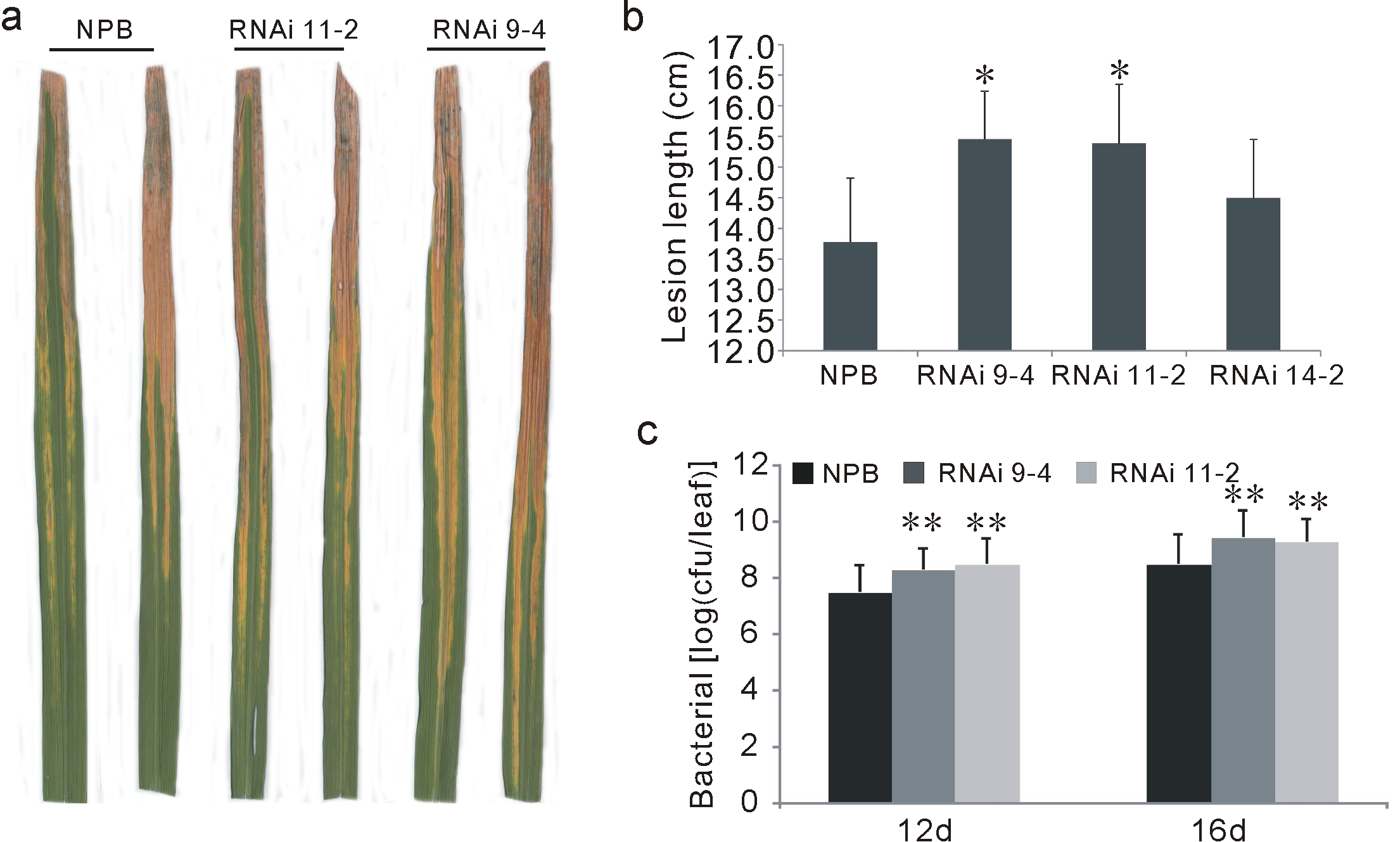

Supplement: Supplementary file 7 — Figure S6. Phenotypes of Nipponbare and OsWRKY67-silenced (WRKY67-RNAi) plants infected with bacterial blight. The values are means ± SDs of twenty biological replicates and the asterisks represent significant differences relative to Nipponbare plants at **P < 0.01 and *P < 0.05 by t-tests. a Disease phenotypes of WRKY67-RNAi and Nipponbare plants after Xoo inoculation. b Lesion lengths in WRKY67-RNAi and Nipponbare plants after Xoo inoculation. c Growth rates of Xoo race 4 in the leaves of WRKY67-RNAi and Nipponbare plants. Bacterial populations were determined from three leaves 12 or 16 days after inoculation by counting colony-forming units (cfu). Similar results were obtained in two independent experiments. (JPG 711 kb) [file 12870_2018_1479_MOESM7_ESM.jpg]

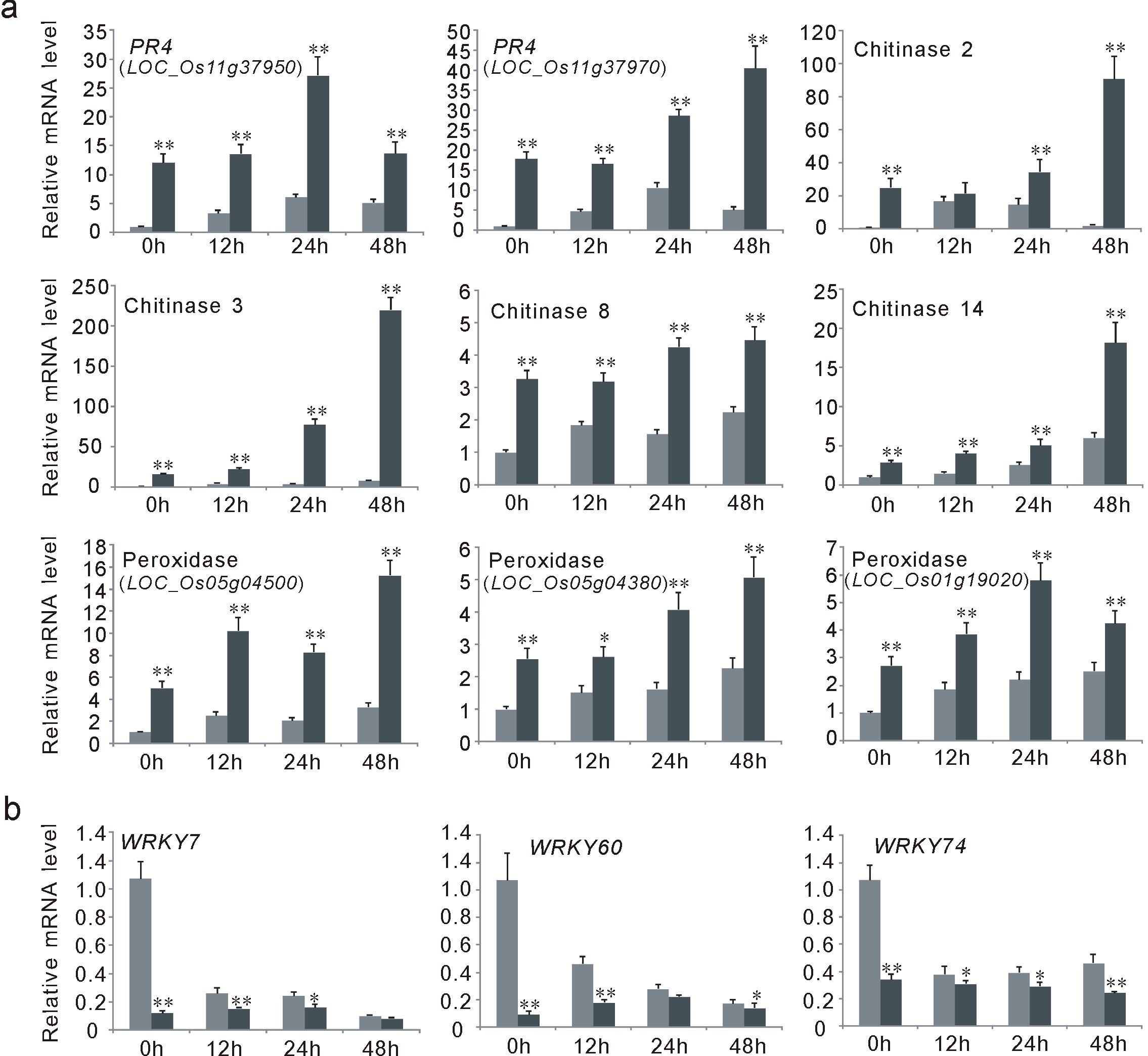

Supplement: Supplementary file 9 — Figure S7. Overexpression of OsWRKY67 influences the expression of defense-related genes. The values are means ± SDs of three biological replicates and asterisks represent significant differences relative to Nipponbare plants before blast inoculation (0 h) or at 12, 24 and 48 h after inoculation with M. oryzae (t-test, **P < 0.01 and *P < 0.05). a Nine defense-related genes were over-presented in OX-WRKY67 plants. b Three WRKY genes were suppressed in OX-WRKY67 plants. (JPG 918 kb) [file 12870_2018_1479_MOESM9_ESM.jpg]

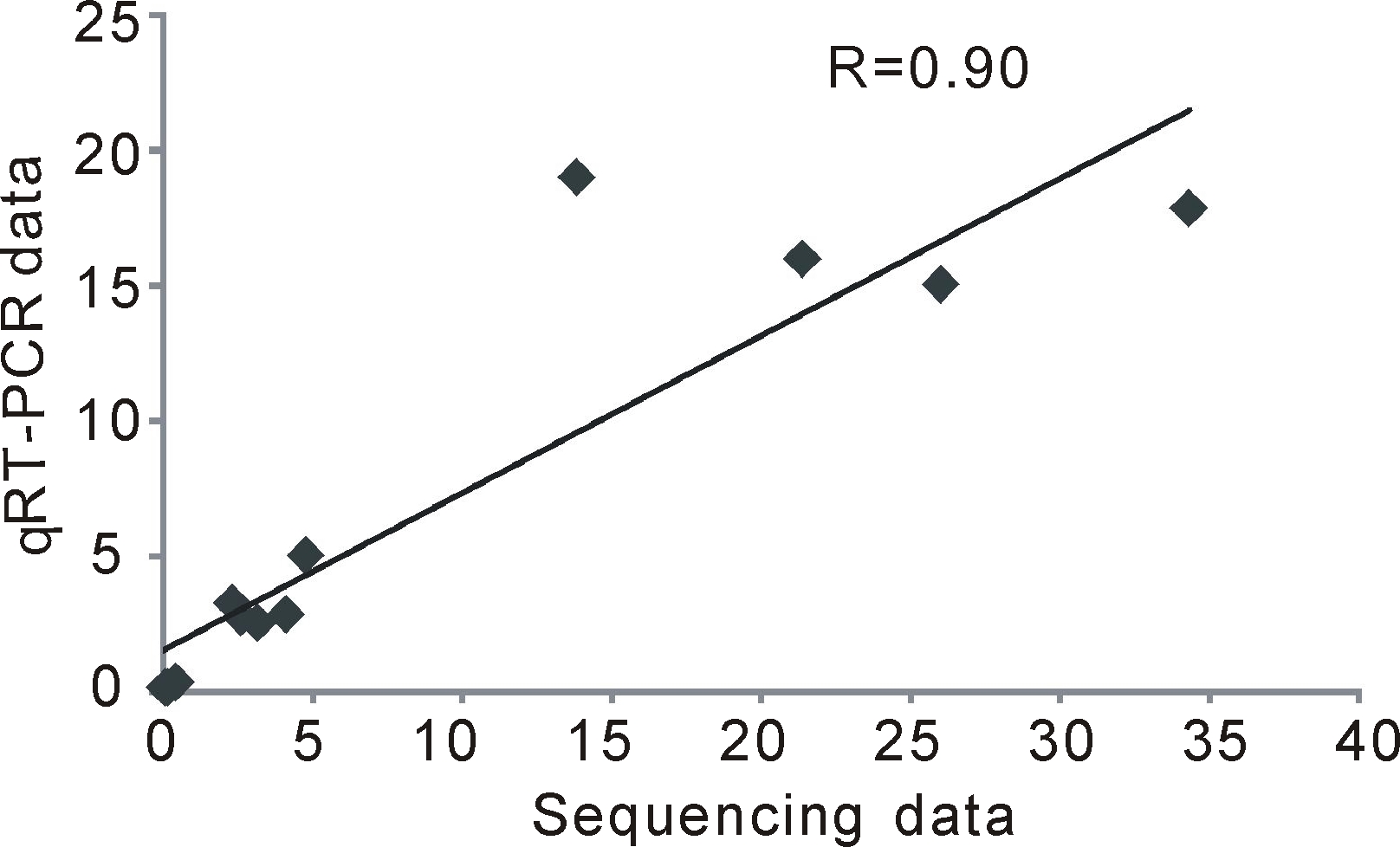

Supplement: Supplementary file 10 — Figure S8. The results of qRT-PCR showed an excellent concordance with the sequencing data. (JPG 214 kb) [file 12870_2018_1479_MOESM10_ESM.jpg]

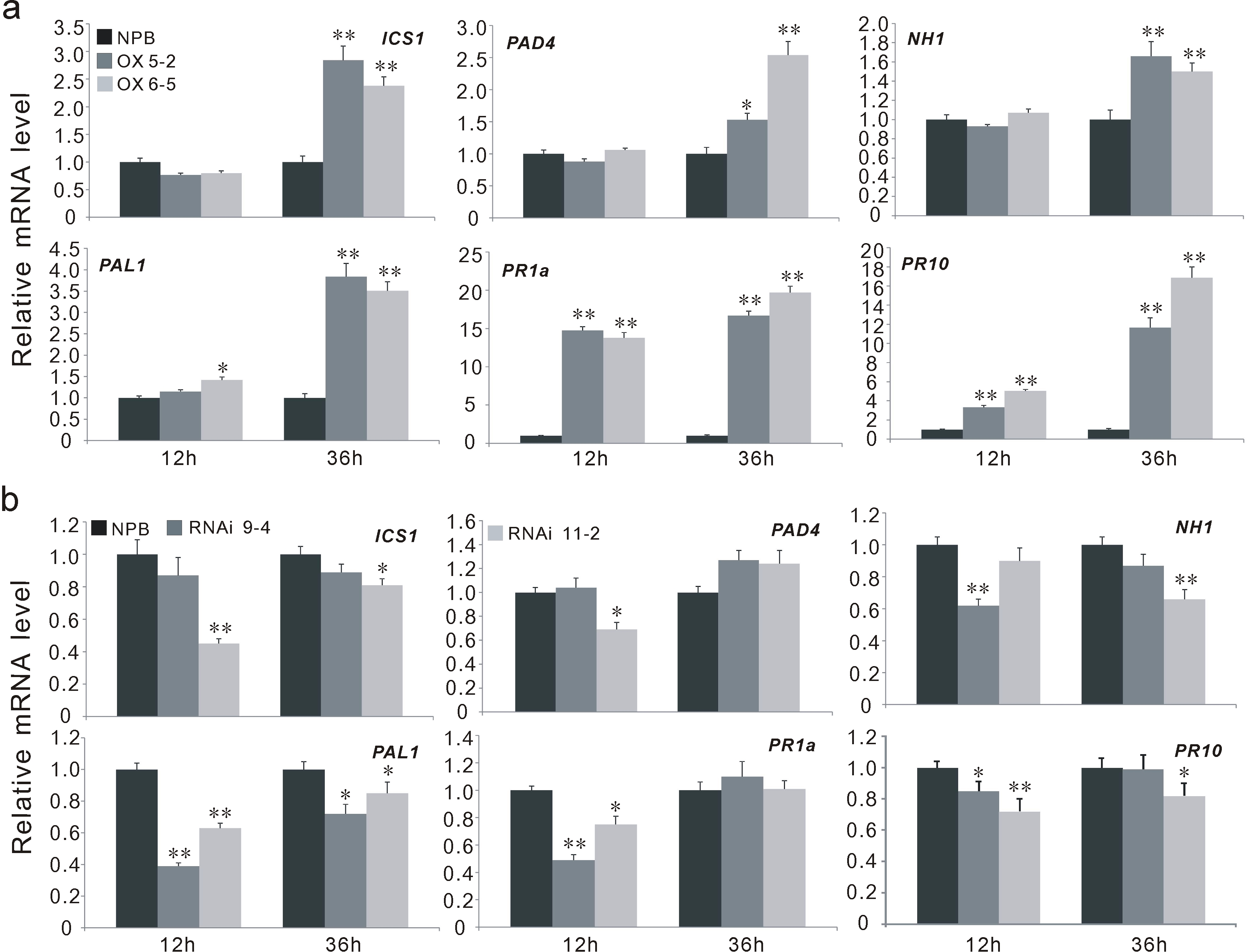

Supplement: Supplementary file 13 — Figure S9. OsWRKY67 regulates the expression of a set of defense-related genes at 12 h and 36 h after bacterial blight infection. The wild-type Nipponbare and transgenic plants were inoculated with Xoo race 4 at the booting stage. The values are means ± SDs of three biological replicates and the asterisks represent significant differences relative to Nipponbare plants at **P < 0.01 or *P < 0.05 by t-tests. The expression of Nipponbare plants was set to “1” at each time point. a Relative expression levels of defense-related genes in Nipponbare and OX-WRKY67 plants. b Relative expression levels of defense-related genes in Nipponbare and WRKY67-RNAi plants. (JPG 1135 kb) [file 12870_2018_1479_MOESM13_ESM.jpg]

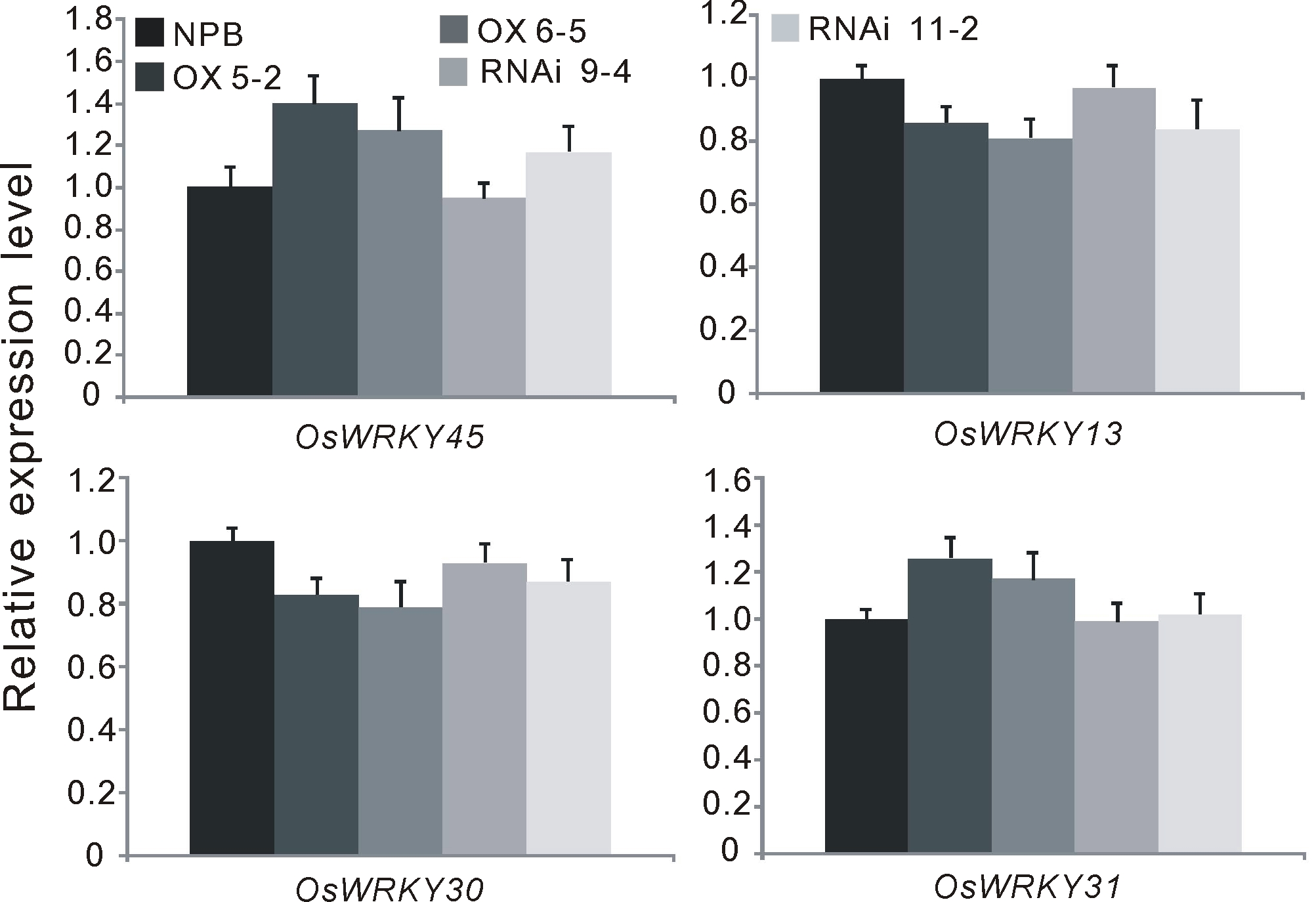

Supplement: Supplementary file 14 — Figure S10. Relative expression levels of four WRKY genes in wild-type Nipponbare and OsWRKY67 transgenic plants. The values are means ± SDs of three biological replicates. (JPG 381 kb) [file 12870_2018_1479_MOESM14_ESM.jpg]
